# Pharmacological modulation of myeloid-derived suppressor cells to dampen inflammation

**DOI:** 10.3389/fimmu.2022.933847

**Published:** 2022-08-30

**Authors:** Chiel van Geffen, Constantin Heiss, Astrid Deißler, Saeed Kolahian

**Affiliations:** Institute of Laboratory Medicine, German Center for Lung Research (DZL), Universities of Giessen and Marburg Lung Center (UGMLC), Philipps University Marburg, Marburg, Germany

**Keywords:** MDSC, pharmacotherapy, immune suppression, immunomodulation, inflammation

## Abstract

Myeloid-derived suppressor cells (MDSCs) are a heterogeneous cell population with potent suppressive and regulative properties. MDSCs’ strong immunosuppressive potential creates new possibilities to treat chronic inflammation and autoimmune diseases or induce tolerance towards transplantation. Here, we summarize and critically discuss different pharmacological approaches which modulate the generation, activation, and recruitment of MDSCs *in vitro* and *in vivo*, and their potential role in future immunosuppressive therapy.

## 1. Introduction

Myeloid-derived suppressor cells (MDSCs) are a heterogeneous immature immune cell population, originating from the common myeloid progenitor cell, with potent immunosuppressive effect on T cell proliferation and activity (1). MDSCs were first described in cancer patients and associated with increased tumor growth and T cell dysfunction (2). In this respect, MDSCs are mainly studied in the tumor microenvironment, where they inhibit the anti-tumor immune response and support tumor angiogenesis (1, 3). In the context of the tumor microenvironment, dampening MDSCs may decrease metastatic niche formation (3–5). However, MDSCs not only play a role in cancer but also in a variety of other pathological conditions associated with an inflammatory state, such as, chronic infections, autoimmunity, asthma, or obesity (6–9). In autoimmune diseases, MDSCs can be useful to protect against tissue damage driven by an imbalanced immune reaction. Furthermore, if the immune response needs to be dampened, e.g. after allograft transplantation, or in conditions such as graft-versus-host disease (GVHD), MDSCs’ immunosuppressive potential might be beneficial. So far, the majority of imbalanced immune conditions are treated by corticosteroids and other immunosuppressive drugs which include substantial side effects. Therefore, MDSCs are a promising therapeutic target due to their immunosuppressive properties (10). This review discusses pharmacological approaches involved in the generation, recruitment and activation of MDSCs, providing possible clues for novel cellular immunosuppressive therapies.

### 1.1 Subsets

Due to the heterogeneity of the MDSC population, most marker proteins are not unique to MDSCs nor universally expressed. Nevertheless, two major subtypes can be distinguished: polymorphonuclear (PMN-) and monocytic (M-) MDSCs based on their similarities to neutrophils and monocytes, respectively. In mice, PMN-MDSCs are defined as CD11b^+^ Ly-6G^+^ Ly-6C^low^ and M-MDSCs as CD11b^+^ Ly-6G^-^ Ly-6C^high^, while in humans PMN-MDSCs express CD11b^+^ CD14^-^ CD15^+^ or CD11b^+^ CD14^-^ CD66b^+^ and M-MDSCs CD11b^+^ CD14^+^ HLA-DR^-/low^ CD15^-^. Only by means of these markers, PMN-MDSCs and M-MDSCs are undistinguishable from neutrophils and monocytes, respectively, and functional assays, such as T cell proliferation and cytokine release assays, are needed to evaluate the immunosuppressive activity (11). Unfortunately, MDSC heterogeneity as well as the significant overlap with more ‘conventional’ immune cell populations complicate the refinement of a universal distinctive MDSC signature. Quantitative tools, such as single-cell RNA sequencing and mass cytometry, as well as other advances contributing to the multi-omics approach, are starting to provide more insights into the phenotypic, morphological and functional heterogeneity of MDSCs. Recently, lectin-type oxidized low-density lipoprotein receptor 1 (LOX-1) has been described as a new marker for PMN-MDSCs in humans. LOX-1 can be found on macrophages, endothelial and smooth muscles cells, but importantly is not expressed on neutrophils and therefore presents a surface marker that helps to identify PMN-MDSCs (12). Furthermore, CD84 and JAML were recently identified as potential markers of MDSCs in breast cancer (13). Additionally, two arginase-1-expressing myeloid clusters were identified –*Spp1^+^Apoe^+^C1qa^+^* and *Gpnmb^+^Vegfa^+^Clec4d^+^Trem2^+^* – in murine tumors, where Trem2 was found to be associated with immunosuppressive function (14).

### 1.2 Origin and generation of MDSCs

During normal hematopoiesis common myeloid progenitor cells develop from hematopoietic stem cells and differentiate *via* immature myeloid cells into red blood cells, granulocytes, monocytes, thrombocytes and mast cells under the influence of growth factors, interleukins and other regulatory molecules. Under certain pathological conditions such as cancer, with chronic stimuli of a relatively low intensity, two signals are required for MDSCs proliferation. The first signal is responsible for stimulation of myelopoiesis with the inhibition of maturation and differentiation of progenitor cells and the expansion of immature myeloid cells while the second signal promotes the transformation into immunosuppressive MDSCs (15). Factors shown to be involved in MDSC expansion and activation include granulocyte-macrophage colony-stimulating factor (GM-CSF), granulocyte colony-stimulating factor (G-CSF), macrophage colony-stimulating factor (M-CSF), stem cell factor (SCF), cyclooxygenase (COX-) 2, prostaglandins (PG), vascular endothelial growth factor (VEGF) and interleukin (IL-) 6 (1). The Janus kinase (JAK) 2/signal transducer and activator of transcription (STAT) 3 pathway appears to be the most important regulator for MDSC expansion (1). For example, GM-CSF, G-CSF, and IL-6 induce the expansion of MDSCs through activation of STAT3 (15–18). The transcription factor interferon regulatory factor (IRF) 8 – downregulated by GM-CSF and G-CSF – acts as a STAT3- and STAT5-dependent negative regulator of MDSC generation (19). In addition, the β_2_-adrenergic receptor (AR) has been shown to play a role in MDSC generation and activation through STAT3 (20). Further underlining the importance of STAT3, several studies revealed that STAT3 inhibition dampened MDSCs in the tumor microenvironment and proved to be a useful anticancer therapy (21–23). Interferon (IFN-) γ activates the STAT1 pathway and leads to activation of MDSCs (24). Similarly, activation of the STAT6 pathway through IL-4 and IL-13 can induce immunosuppressive properties of MDSCs (15). Pro-inflammatory mediators such as tumor necrosis factor (TNF-) α, IL-1β, IL-12, PGE_2_ or Toll-like receptor (TLR) ligands enhance the immunosuppressive capacities of MDSCs through the nuclear factor kappa-light-chain-enhancer of activated B cells (NF-κB) pathway (25–30). In detail, activation of PGE_2_ receptor (EP) 2 and EP4 inhibits receptor-interacting protein kinase 3 (RIPK3), which in turn enhances the NF-κB pathway, as activated RIPK3 is involved in its downregulation (31). Furthermore, the endoplasmic reticulum (ER) stress response pathway ends up in the NF-κB pathway and promotes the activation of immunosuppressive MDSCs. The function of this pathway is to protect the cell from cellular stress like shortage of nutrients, hypoxia, or low pH (12). Furthermore, inhibition of the Notch pathway and activation of the adenosine receptor promote the expansion of MDSCs (32, 33). Those mechanisms create possible targets for *in vitro* and *in vivo* generation of MDSCs. MDSCs themselves are considered as immature cells with a high plasticity. Under hypoxic conditions, MDSCs are able to differentiate into tumor-associated macrophages, M2-like macrophages, inflammatory dendritic cells or fibrocytes (34). Hence, some researchers state that MDSCs are not a definitive cell group, but rather cells in transitory states, whose differentiation is ongoing.

### 1.3 Recruitment

Once generated, MDSCs are recruited to the site of activity by chemokines. The role of several cytokines, chemokines and their receptors in MDSC recruitment is reviewed elsewhere (35). These chemokines have been described to play an important role in MDSC recruitment through their interaction with corresponding G-protein coupled chemokine receptors: C-X-C motif ligand (CXCL)1/CXCL2/CXCL5 with C-X-C motif receptor (CXCR)2, CXCL8 with CXCR1/CXCR2, CXCL17 with CXCR8, C-C motif ligand (CCL)2/CCL12 with CCR2, CCL3/CCL4/CCL5 with CCR5, and CCL15 with CCR1. Specifically, CXCR2 appears to be critical for MDSC recruitment (4, 36–42).

### 1.4 Mechanisms of immunosuppressive activity

Activated MDSCs carry out their suppressive activity on T cell proliferation by different mechanisms, which were reviewed by Gabrilovich and Nagaraj (1). Upon upregulation of transcription factors like STAT1, 3, 6 and NF-κB, the expression of reactive oxygen species (ROS), arginase-1 (Arg-1), inducible nitric oxide synthase (iNOS), NF-κB and idoleamine 2,3-dioxygenase (IDO) is increased. Production of ROS by nicotiamide adenine dinucleotide phosphate (NADPH) oxidase suppresses T cell function by destroying proteins, lipids and inducing apoptosis among other mechanisms (43, 44). Both Arg-1 and iNOS deprive L-arginine – an amino acid essential for T cell metabolism – from the microenvironment and thus, inhibit T cell proliferation by suppressing T cell cycle progression (45). In addition, the iNOS product nitric oxide (NO) suppresses T cell function and induces apoptosis by itself through different mechanisms (1). Simultaneously, IDO inhibits T cell proliferation by depleting tryptophan from T cell metabolism as well as increasing regulatory T cells (Treg) recruitment (46). Shifting the T cell population towards immunosuppressive forkhead box (Fox) P3^+^ Tregs presents another effective way of immunosuppression (47, 48). In addition, MDSCs have been shown to suppress B cell responses (49). There are also subset specific differences: M-MDSCs mainly use NO produced by iNOS to suppress T cell function, while PMN-MDSCs express higher levels of ROS and peroxynitrite, a product from the reaction of NO and superoxide anion (50, 51). Both M- and PMN-MDSC secrete Arg-1 (50).

## 2. Pharmacological approaches to modulate MDSCs

**Figure 1:**
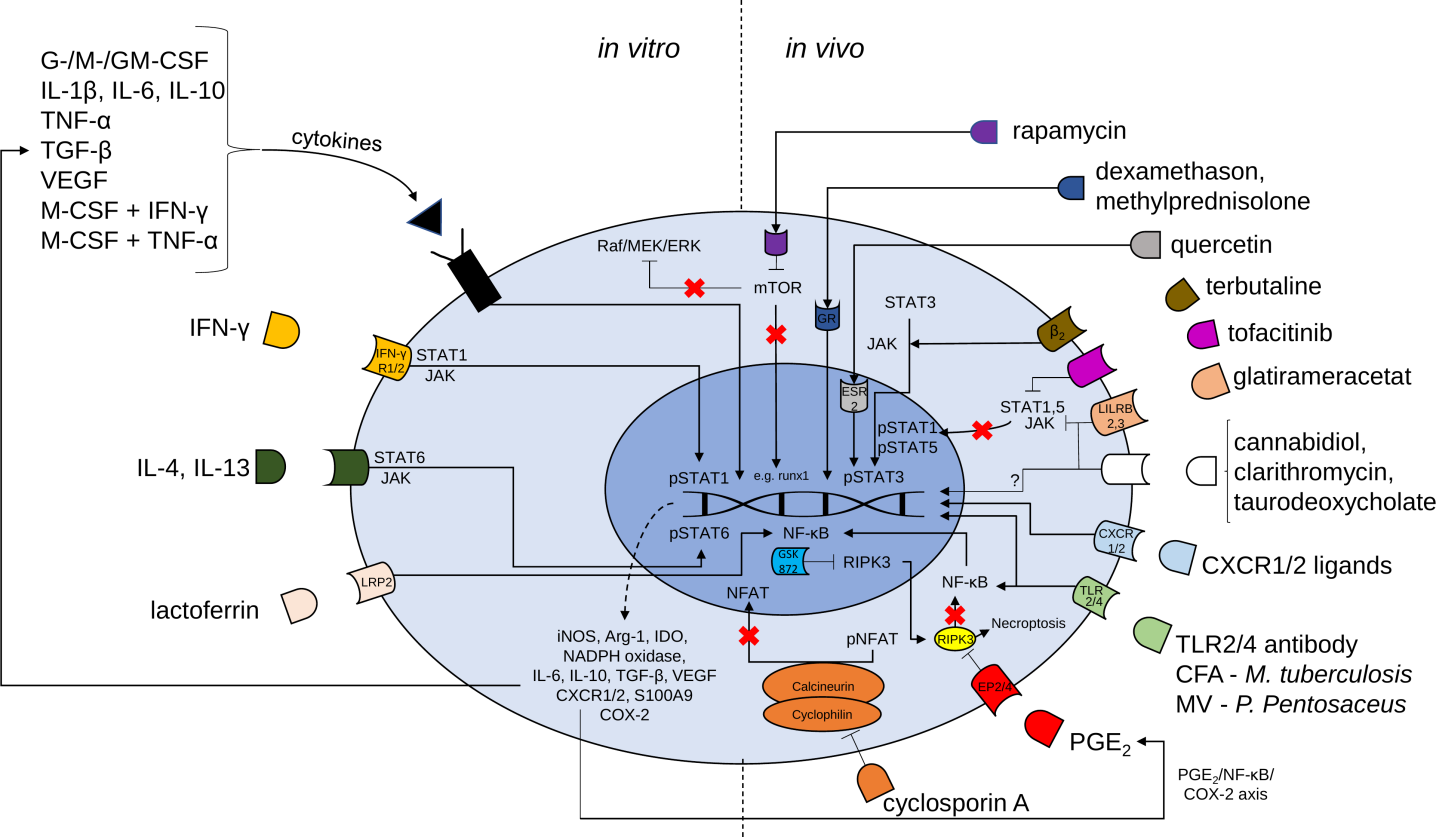
Mechanisms of possible pharmacological targets for the induction of MDSCs. Proliferation and activation of MDSCs is regulated by transcription factors such as signal transducer and activator of transcription (STAT) 3, STAT1, STAT5, STAT6 and nuclear factor kappa-light-chain-enhancer of activated B cells (NF-κB). *In vitro* generation of MDSCs is induced by a large variety of cytokines and cytokine combinations and lactoferrin. MDSC generation may be followed by adoptive transfer cell therapy to induce the beneficial effects of MDSCs. Various compounds [e.g. rapamycin, glucocorticoids, terbutaline, tofacitinib, glatirameracetat, cannabidiol, clarithromycin, taurodeoxycholate, CXCR1 or CXCR2 ligands, complete Freund’s adjuvant (CFA), membrane vesicles (MV), Toll-like receptor (TLR) 2/4 agonistic antibodies, prostaglandin E_2_, cyclosporine A and receptor interacting protein kinase (RIPK) 3 inhibitor (GSK 872)] may be used for accumulation of MDSCs *in vivo*. *G-CSF* granulocyte colony-stimulating factor, *M-CSF* macrophage CSF, *GM-CSF* granulocyte-macrophage CSF, *IL* Interleukin, *TNF* tumor necrosis factor, *TGF* transforming growth factor, *VEGF* vascular endothelial growth factor, *IFN* interferon, *mTOR* mammalian target of rapamycin, *JAK* Janus kinase, *LRP* lactoferrin receptor, *iNOS* inducible nitric oxide synthase, *Arg* arginase, *IDO* indoleamine 2,3-dioxygenase, *NADPH* nicotiamide adenine dinucleotide phosphate, *NFAT* nuclear factor of activated t cells, *p* phosphorylated, *CXCR* CXC chemokine receptors, *COX* cyclooxygenase.

There are in principle two main pharmacological approaches for MDSC generation: Expanding MDSCs *ex vivo* and adoptively transferring them into patients or stimulating endogenous MDSC expansion/activation. Possible pharmacological approaches for MDSCs generation, activation and recruitment are presented in **Table 1** as well as **Figure 1**.

**Table 1:** Potential pharmacological targets and drugs to modulate MDSCs to dampen inflammation.

Target	Potential pharmacological drug	Effect of potential drug on MDSCs	Murine model(s)	Reference
β2-AR	β2-AR agonists (Terbutalin)	Increased number	GVHD	(52)
Calcineurin	Calcineurin inhibitors (Cyclosporin A, Tacrolimus)	Increased number Increased activity	Skin allograft	(53, 54)
CXCR1,2	CXCR1, CXCR2, CXCL17 agonists	Increased number Increased activity	Pulmonary hypertension	(4, 36–40)
EP2/4	PGE2 (EP2/4 agonists)	Increased number Increased activity	Asthma	(31, 55–57)
ERK1/2	Glucosamine	Increased number Increased activity	–	(58)
ESR2	Quercetin	Increased number Increased activity	Prostate carcinoma	(59, 60)
ETAR	ETAR antagonists (BQ123)	Increased number	Autoimmune hepatitis Colitis Pneumonia	(61, 62)
Glucocorticoid receptor	Glucocorticoids (Dexamethasone, Methylprednisolone)	Increased number	Cardiac/skin allograft Multiple sclerosis	(63–65)
LILRB	Glatirameracetat	Increased number Increased activity	Inflammatory bowel disease	(66, 67)
LRP2	Lactoferrin	Increased number	Autoimmune hepatitis Lung inflammation Necrotizing enterocolitis	(68)
mTOR	mTOR inhibitors (Rapamycin)	Increased number Increased activity	Cardiac/corneal/skin allograft GVHD Heart failure Hepatic/renal injury Wound healing	(40, 48, 69–77)
RIPK3	RIPK3 inhibitors (GSK872)	Increased number	Autoimmune hepatitis Multiple sclerosis	(78–80)
STAT1, STAT5	Tofacitinib, IFN-γ	Increased number Increased activity	Arthritis Interstitial lung disease	(81, 82)
TLR2, TLR4	TLR2 ligands, TLR4 ligands (CFA-M.tuberculosis, MV-P.pentosaceus)	Increased number Increased activity	Fibrosis Peritonitis Type-1-diabetes	(83–87)
Unclear	Cannabidiol	Increased number	Autoimmune hepatitis Multiple sclerosis	(88, 89)
Unclear	Claritromycin	Increased number	Post-influenza pneumonia Sepsis	(90)
Unclear	Taurodeoxycholate	Increased number Increased activity	Sepsis	(91)

β2-AR, β2-adrenergic receptor; CFA, Complete Freund’s adjuvant; CXCR, C-X-C chemokine receptor; EP, Prostaglandin E2 receptor; ERK, Extracellular signal-regulated kinase; ESR, Estrogen signaling receptor; ETAR, Endothelin A receptor; GVHD, Graft-versus-host disease; LILRB, Leukocyte immunoglobulin-like receptor B; LPS, Lipopolysaccharide; LRP, Lactoferrin receptor; mTOR, Mammalian target of rapamycin; MV, Membrane vesicles; RIPK3, Receptor-interacting protein kinase 3; STAT, Signal transducer and activator of transcription; TLR, Toll-like receptor.

### 2.1 Adoptive transfer of *in vitro/ex vivo* generated/activated MDSCs

Non-stem cell-based cell therapies that use cells such as CAR-T cells, dendritic cells and natural killer cells are promising and rapidly evolving (92). MDSC cell therapy may be another promising strategy in this list, especially considering its potent immunosuppressive capacity and its role in maintaining immune tolerance in transplantation and autoimmunity. Several pre-clinical studies have shown promising therapeutic effects of the adoptive transfer of MDSCs in organ transplantation, autoimmune diseases as well as in a variety of other immune-related disorders, such as cyclosporin A-induced hypertension, heart failure and asthma (63, 69, 93–100). Here, we briefly discuss some of the cytokines and pharmacological compounds that have been identified to promote the generation and/or activation of MDSCs *in vitro*/*ex vivo* and were applied as MDSC cell therapy.

#### 2.1.1 Cytokines

Park et al. demonstrated the beneficial role of *in vitro* generated MDSCs in the context of GVHD (101). Here, the highest efficiency of *in vitro* production of MDSCs from CD34^+^ human umbilical cord blood cells was achieved with a combination of GM-CSF and SCF, whereas G-CSF/SCF and M-CSF/SCF were less effective (101). Adoptively transferred MDSCs were shown to ameliorate GVHD and prolong survival in a murine xenogeneic model of GVHD by promoting Tregs and inhibiting the T helper (Th) 1 and Th17-driven inflammatory responses (101). MDSCs generated with GM-CSF/SCF were shown to exhibit increased immunosuppressive activity after the transfer *in vivo* compared to MDSCs generated in the presence of either G-CSF/SCF or M-CSF/SCF (101). Hsieh et al. also showed beneficial effects of adoptive transfer of MDSCs in renal fibrosis and diabetic neuropathy in diabetic mice (102). Here, murine bone marrow-derived MDSCs were induced with GM-CSF, IL-1β and IL-6 *in vitro*. In addition, Yang et al. induced M-MDSCs with combinations of M-CSF/IFN-γ and M-CSF/TNF-α, respectively, and demonstrated prolonged skin allograft survival upon adoptive transfer (103, 104). *In vitro* generated MDSCs, induced by GM-CSF, G-CSF, and IL-6, efficiently ameliorated autoimmune arthritis in mice (105). Recently, another group demonstrated that the adoptive transfer of splenic CD11b^+^Gr-1^+^ MDSCs, obtained from G-CSF-treated donor mice, are capable of prolonging heart allograft survival (93).

As already discussed above, there are many cytokines and cytokine combinations identified to play important roles in MDSC generation and activation. Here it is important to consider that certain cytokine signaling is required to induce MDSCs, and the discussed pharmacological compounds are insufficient to induce *in vitro*/*ex vivo* MDSCs without additional cytokine signaling.

#### 2.1.2 Glucocorticoids

Zhao et al. induced MDSCs *in vitro* by means of GM-CSF and dexamethasone (63). Adoptive transfer of dexamethasone-induced MDSCs into mice prolonged heart allograft survival, likely through increased levels of iNOS and increased number of Tregs (63). Indeed, dexamethasone is already an established immunosuppressive drug, and these findings show that a part of its immunosuppressive activity is likely mediated by their effect on MDSCs.

#### 2.1.3 Lactoferrin

Adoptive transfer of *in vitro* generated murine bone marrow-derived MDSCs treated with lactoferrin (LF) prolonged survival and ameliorated inflammation in necrotizing enterocolitis in newborn mice as well as concanavalin-induced hepatitis and ovalbumin-induced lung inflammation (68). Both *in vitro* and *in vivo* LF treatment increased MDSC numbers, likely *via* NF-κB activation, but only in infant mice due to decreased LF receptor (LRP) 2 expression in adults (68). However, *in vivo* administration of LF was shown to be much less effective in recruiting and activating MDSCs compared to the adoptive transfer of *in vitro* generated MDSCs using LF. This study demonstrates the potential of targeting NF-κB and LRP2 to recruit MDSCs (68).

#### 2.1.4 PGE_2_

PGE_2_, in combination with GM-CSF and either IL-4 or IL-6, was found to efficiently induce MDSCs *ex vivo* (55, 100). From the four EP subreceptors (EP1-4) of PGE_2_, EP2 and EP4, and not EP1 and EP3, were found to induce MDSC development, hinting at an important role of the adenylate cyclase/cAMP/PKA/CREB signaling pathway (55, 100). Furthermore, the adoptive transfer of MDSCs generated in the presence of a selective EP4 receptor agonist dampened airway inflammatory features in a murine model of asthma (100).

#### 2.1.5 Phorbol 12-myristate 13-acetate

The combination of M-CSF and PMA was recently shown to induce MDSCs *in vitro* (106). The adoptive transfer of PMA-induced MDSCs induced immune tolerance in a mouse skin transplantation model, by inhibiting the T cell response, promotion of cytokine secretion and inducing Tregs (106). PMA significantly upregulated Arg-1 expression in MDSCs, and an Arg-1 inhibitor (nor-NOHA) diminished MDSC activity (106). This study confirms that *in vitro* induced-MDSCs may be promising targets for adoptive transfer to modulate immunosuppression, such as in organ transplantation.

#### 2.1.6 Rapamycin

The mTOR inhibitor rapamycin is frequently used in immunosuppressive therapy following allograft transplantation in order to prevent allograft rejection. Rapamycin is also applied on drug-eluting stents to decrease stent stenosis, *via* its anti-proliferative properties. Nakamura et al. performed adoptive transfer of *in vitro* generated MDSCs, treated with rapamycin, directly into the coronary artery of heart allografts in mice (48). Administration of rapamycin-induced MDSCs prolonged skin allograft survival and demonstrated the possibility of local MDSC therapy. Adoptive transfer of rapamycin-treated MDSCs resulted in improved outcomes concerning acute kidney injury, with increased Tregs number and decreased pro-inflammatory cytokines compared to adoptive transfer of MDSCs not treated with rapamycin (40).

To this end, the *in vitro/ex vivo* generation and subsequent adoptive transfer of MDSCs has been established in animal models and most studies observe a beneficial effect in the treatment of inflammatory diseases, yet human studies are needed to further confirm the therapeutic concepts. However, at the time of writing this review, no clinical trials investigating MDSC adoptive transfer as a cell therapy have been performed nor were registered in the clinical trial databases of the National Institute of Health^1^ or the EU clinical trial register^2^. Nevertheless, the generation/expansion of endogenous MDSC, by administration of the right cytokine combination or potential medications, would be the more elegant way to generate, activate and recruit the MDSCs and dampen inflammatory diseases. We discuss the possibilities in the following chapter of this review.

### 2.2 *In vivo* generation, recruitment, and activation of MDSCs

#### 2.2.1 Acetaminophen

Hsu et al. showed that the increase of intrahepatic MDSCs by a sublethal dose of acetaminophen was able to protect mice against subsequent lethal doses of acetaminophen, lipopolysaccharide (LPS)/D-galactosamine or concanavalin A (107). This finding was confirmed by a loss of protection after MDSC depletion (107). The observed protective effect was likely mediated by increased iNOS expression, as iNOS-expressing MDSCs were found to induce the apoptosis of activated neutrophils and decreased the intrahepatic infiltration of elastase-expressing neutrophils (107). Furthermore, the group of Hsu et al. were able to generate human PMBC-derived MDSCs with a similar phenotype (107).

#### 2.2.2 Rapamycin and other mammalian target of rapamycin inhibitors

Besides the immune regulating features of rapamycin and its ability to generate MDSCs *in vitro* for cell therapy, rapamycin has also been linked to MDSC recruitment and activation *in vivo*. Nakamura et al. reported prolonged heart allograft survival in mice under Rapamycin administration, which was found to be related to increased number of MDSCs, particularly M-MDSCs, and iNOS expression (48). Zhang et al. showed that rapamycin treatment ameliorated acute kidney injury through the recruitment and activation of mainly PMN-MDSCs (40). A recent study by Scheurer et al. demonstrated that rapamycin administered after bone marrow transplantation promoted immunosuppressive properties of MDSCs and thus prevented GVHD (70). Furthermore, rapamycin treatment ameliorated heart failure in mice, likely through the induction of MDSCs, as MDSC depletion diminished this beneficial effect (69). Wei et al. treated cornea transplanted mice with eye drops containing rapamycin nano-micelles (71). An increased recruitment of MDSCs and expression of Arg-1 and iNOS was observed and was in line with a prolonged allograft survival. Also, in immunological hepatic injury, rapamycin treatment was found to promote MDSC recruitment, generation, and activity, and ameliorated the disease (72). Recently, our group demonstrated the allograft survival prolonging properties of rapamycin in obese mice through increased M-MDSCs number and activity (73). Taken together, rapamycin seems to be an efficient inductor of MDSC generation and activation, with the advantage of already being approved for clinical use for certain diseases. Furthermore, a novel mTOR inhibitor, INK128, was shown to promote wound healing in streptozotocin-induced diabetic mice (74). mTOR deficiency in M-MDSCs was shown to induce tolerance of mouse cardiac allografts (75), while adoptive transfer of PMN-MDSCs lacking mTOR expression was shown to dampen acute GVHD (76), confirming the beneficial role of mTOR inhibition in promoting MDSC immune suppression.

However, the linking mechanism between mTOR and MDSCs is not fully understood. Nakamura et al. stated that mTOR inhibition could lead to increased activation of the Raf/MEK/ERK pathway, which caused MDSC recruitment and activation (48). Another possible mechanism responsible for MDSC activation is the downregulation of runt-related transcription factor 1 (*runx1*) gene expression (40). A third possible mechanism was recently described by Jia et al. who reported that AKT1, a known downstream target of mTOR, regulates MDSC immunosuppressive activities by suppressing hypoxia-inducible factor 1α-dependent glycolysis (77). Therefore, further research is necessary to further clarify the underlying mechanism.

#### 2.2.3 Glucocorticoids

Evidence emerged showing that glucocorticoids may also carry out their immunosuppressive effect through the induction as well as the recruitment of MDSCs. Mechanistically, the glucocorticoid receptor (GR) is critical for the immunosuppressive activity of MDSCs as GR activation leads to a release of CXCR2, which is one of the main chemokines involved in the recruitment of MDSCs to areas of inflammation (64). Liao et al. demonstrated the same effects and mechanisms of dexamethasone administration *in vivo* in a mouse model of skin allograft transplantation (64). Another frequently used glucocorticoid is methylprednisolone. Direct correlations between methylprednisolone administration and increases in the number of MDSCs, particularly PMN-MDSCs, have recently been demonstrated in a murine model of multiple sclerosis (MS) and human MS patients by Wang et al. (65). Interestingly, a difference in MDSC immunosuppressive activity was observed in mice compared to human MS patients, whereas MDSCs show higher immunosuppressive activity in the latter upon treatment with methylprednisolone. This was revealed by measuring MDSC activity in experimental autoimmune encephalomyelitis (EAE) mice and MS patients, before and after methylprednisolone treatment (65). In mice, increased number of MDSCs were observed at the onset of EAE, but not after methylprednisolone pulse therapy. In contrast, in MS patients increasing MDSC numbers were observed in PBMCs after methylprednisolone application, and disease remission after treatment was correlated to the increased number of MDSCs (65). This highlights the complexity and importance of analyzing MSDC behavior, not only in mice, but also in humans. Furthermore, the mechanism of MDSC induction by glucocorticoids is linked to inhibition and downregulation of GR-β, which, if activated, antagonizes the effect of glucocorticoids (65). Mice only express one type of GR which might explain the higher MDSC activity observed in humans (63). Methylprednisolone is considered the main drug for chronic inflammatory conditions and is frequently used, not only in MS patients, but also in many other autoimmune or chronic inflammatory diseases like chronic obstructive pulmonary disease (COPD) and rheumatic diseases (108). Hence, methylprednisolone and the GR, with its different subtypes, may provide promising targets for further research.

#### 2.2.4 Chemokines

CXCR1 and CXCR2, among others, are highly expressed on MDSCs and responsible for their recruitment. Blockage of CXCR1 and CXCR2 with selective antagonists was shown to severely decrease the number of MDSC infiltration in pulmonary hypertension and carcinomas (4, 37). Furthermore, intratracheal administration of recombinant mouse protein CXCL17 has been shown to result in the recruitment of MDSCs into the lungs of mice (36). Selective CXCR1, CXCR2 and CXCL17 agonists seem to be promising pharmacological drugs for increasing MDSC migration. However, these agonists need to be further studied in the context of MDSC recruitment. Recently, 2,3,7,8-tetrachlorodibenzo-*p*-dioxin (TCDD) – an environmental pollutant and aryl hydrocarbon receptor (AhR) agonist – was shown to indirectly recruit MDSCs through strong induction of CXCR2 expression by down-regulating specific miRNAs (39). Upregulation of transcription factors responsible for CXCR2 ligands (CXCL1 and CXCL2) expression, like Snail, which acts through NF-κB pathway, also provides other possible targets related to chemokines (38). In addition, mTOR inhibition with rapamycin was found to result in increased CXCR2, CXCL1 and CXCL2 expression, which was directly linked to MDSC recruitment to the site of acute kidney injury (40). Overall, targeting chemokines and their receptors for MDSC recruitment seems promising, but the involved risks, as previously reported in cancer patients, should be taken into careful consideration (3–5). However, MDSCs primarily are involved in promoting pre-metastatic niche formation and not directly in tumor growth itself (109). Thus, the question can be raised whether it could be beneficial to use locally administered CXCR1 and CXCR2 ligands or CXCL17 only for tumor-free patients in sites of chronic inflammation. As administration of those ligands could increase the risk of pre-metastatic niche formation if tumor cells are present, but not if the patient is tumor-free. Alternatively, coating of allografts with chemokines and cytokines, e.g. GM-CSF and CXCL17, could be used to recruit MDCS to the allograft and locally reduce the immune response.

#### 2.2.5 Endothelin (ET)_A_ receptor antagonist

ET signaling mediates strong vasoconstrictor properties, and the ET_A_ receptor antagonist, BQ123, is an effective therapy for hypertension and obese cardiomyopathy (61). Recently, BQ123 was shown to induce PMN-MDSC-mediated immune suppression in dextran sulfate sodium-induced colitis, papain-induced pneumonia, and concanavalin A-induced hepatitis in mice (62). Both the treatment of BQ123, as well as the transfer of BQ123-induced PMN-MDSCs were effective in dampening inflammation (62). Further analysis showed that BQ123 mediates it effects through the IL13/STAT6/Arg1 signaling pathway (62).

#### 2.2.6 β_2_-agonists

Recently, one promising approach in endogenous MDSC generation was achieved with the help of β_2_-AR agonists in a murine model of GVHD (52). Treatment with bambuterol – a prodrug of the selective β_2_-agonist terbutaline – significantly increased the number of MDSCs and Tregs while effector T cells were reduced and GVHD was ameliorated (52). All other mechanisms of action were excluded, and the central role of β_2_-AR was confirmed (52). Accordingly, *in vitro* cultivation of MDSCs is also increased by terbutaline (52). Terbutaline and other β_2_-agonists are used to treat asthma or COPD due to their bronchodilator effect. However, part of their beneficial role also might originate from MDSC generation.

#### 2.2.7 TLR ligands

TLR4 signaling has been shown to play a crucial role in inducing MDSCs (83). In a study on acute type 1 diabetes using non-obese diabetic (NOD) mice, an agonistic TLR4 monoclonal antibody was found to have a protective effect through the induction of MDSCs (84). Furthermore, the adoptive transfer of *ex vivo* bone marrow cells, stimulated with TLR4 antibody, into NOD mice suppressed acute type 1 diabetes induction as well (84). TLR4 antibody was shown to alter TLR4 signaling, including NFκB signaling, resulting in the downregulation of inflammatory genes and proteins (84). Furthermore, TLR2 activation by Pam2CSK4 was found to enhance immunosuppressive activity of M-MDSCs by upregulating iNOS and NO production, partly through STAT3 activation (85). Successful *in vivo* generation of MDSCs was also achieved with administration of complete freund’s adjuvant (CFA) containing heat-killed *Mycobacterium (M.) tuberculosis* in mice (86). Interestingly, a single dose of CFA increased the number of MDSCs but did not increase MDSC activity. However, a second dose of CFA was shown to increase MDSC activity as well. Here, M-MDSC accumulation and activation was found to be favored. *M. tuberculosis* was shown to play a pivotal role in the induction of MDSC accumulation by CFA, most likely through activation of TLR pathways such as TLR2 and TLR4 (86). Efficient MDSC boosting is also achieved using isolated membrane vesicles (MVs) of the common human gut bacterium P. pentosaceus *via* the TLR2 pathway (87). Alpdundar Bulut et al. observed upregulation of MDSC numbers, Arg-1 and IL-10 levels and M2-like macrophage differentiation *in vitro* as well as in several murine models modelling different inflammatory conditions (87). Isolated MV administration was shown to result in improved disease outcome (87). As the role of TLRs in MDSC induction is well documented, it appears to be a logical pharmacological target with potentially promising effects of TLR2/4 antibodies and/or agonists in promoting MDSCs and dampening inflammation (83).

#### 2.2.8 PGE_2_/RIPK3 inhibitors

Increased PGE_2_ levels resulted in elevated activity and production of Arg-1, IL-6, VEGF, S100A9 and NO, while it also further inhibited RIPK3, creating a positive feedback loop for MDSC activation (31). Decreased receptor-interacting protein kinase 3 (RIPK3) expression was found to be correlated with increased MDSC number and activity in colorectal and hepatocellular carcinoma, likely mediated by the NF-κB/COX-2/PGE_2_ axis (31, 78). Inhibition of RIPK3 with GSK872 was shown to prevent immune-mediated hepatitis (79). The authors showed that RIPK3 inhibition led to an increase in MDSCs number, which likely mediated the observed protective effect, as it was lost after MDSC depletion (79). RIPK3 knockdown resulted in increased MDSC recruitment in a mouse model of hepatocellular carcinoma, which could be inhibited by a CXCR2 antagonist (78). All in all, these findings indicate that RIPK3 inhibition may be another promising pharmacological target to promote MDSC recruitment, likely *via* the NF-κB/COX-2/PGE_2_ and CXCR2 chemokine axis. Another possible MDSC activation mechanism linked to the NF-κB/COX-2/PGE_2_ axis may be the target of the PGE_2_ receptors EP2 and EP4. Both EP2 and EP4 receptors may be promising targets for research by inducing them *via* their transcription factors or activating them with agonists. The study of Jontvedt Jorgensen et al. highlighted the potential of using this axis as a therapeutical target, as they demonstrated reduced MDSC numbers in murine models of tuberculosis after COX-2 inhibitor administration (56). Recently, we showed that a selective EP4 agonist could generate and activate MDSCs and dampen airway inflammation in a murine model of asthma (57).

#### 2.2.9 Tofacitinib

Tofacitinib is a JAK inhibitor approved for treatment of rheumatoid arthritis (RA), among others. Sendo et al. showed that tofacitinib promoted MDSC expansion and ameliorated chronic inflammation in a murine model of RA-associated interstitial lung disease (ILD) (81). Previously, the same group showed the increase of the number of MDSCs and the following improvement of RA upon tofacitinib administration (82). Tofacitinib treatment was found to inhibit phosphorylation of STAT1 and STAT5, while STAT3 levels remained constant which underlined the role of STAT3 in MDSC activation. The beneficial effect of tofacitinib in ILD may reveal the potential effect of JAK inhibitors on MDSC recruitment.

#### 2.2.10 Quercetin

Quercetin, a natural substance found in many fruits and seeds, has already been shown to have anti-inflammatory properties in the context of autoimmune diseases such as rheumatoid arthritis and inflammatory bowel disease (110–112). Furthermore, Quercetin has been used as an anti-tumor therapy, due to its potential to induce tumor apoptosis and necrosis (59). Recently, Ma et al. showed a controversial correlation between MDSC regulation through quercetin and its anticancer properties (60). Quercetin treatment promoted PMN-MDSC expansion as well as its immunosuppressive activity in a murine model of prostate cancer (60). Mechanistically, quercetin binds to estrogen signaling receptors (ESR), especially ESR2, and exerts downstream phosphorylation of STAT3 in PMN-MDSCs and increased the expression of iNOS, NADPH oxidase and IDO. However, the same study showed that quercetin induces apoptosis in M-MDSCs through the estrogen signaling pathway (60). Nevertheless, quercetin may be a promising compound for the selective induction and activation of PMN-MDSCs through ESR/STAT3 signaling pathway, further supplementing the anti-inflammatory properties associated with quercetin.

#### 2.2.11 Cannabidiol

Recently, Elliott et al. showed that administration of cannabidiol – a non-psychoactive cannabinoid – ameliorated autoimmune encephalomyelitis in mice through the induction of MDSCs (88). Adoptive transfer of *in vitro* generated MDSCs, treated with CBD, was similarly found to improve encephalomyelitis, while MDSC depletion had the opposite effect. The same group demonstrated similar effects of CBD treatment in autoimmune hepatitis induced mice (89).

#### 2.2.12 Clarithromycin

One study also revealed clarithromycin as a potential target for MDSC induction *in vivo* (90). Intraperitoneal and oral treatment with clarithromycin was shown to increase the number of MDSCs and prolong their survival in a murine model of LPS endotoxin shock and post-influenza pneumococcal pneumonia. In healthy humans, clarithromycin intake seems to enhance immunosuppressive activity of MDSCs (90).

#### 2.2.13 Taurodeoxycholate

Chang, S. et al. indicated that TDCA – a taurine-conjugated bile acid – can be used to induce MDSCs (91). Here, administration of TDCA was found to improve survival in a mouse model of sepsis, likely through the generation and activation of MDSCs. Still, further research is needed to better understand the underlying mechanisms.

#### 2.2.14 Cyclosporin A

Calcineurin inhibitors such as cyclosporin A or tacrolimus/FK506 are commonly used to treat autoimmune diseases and are administered to allograft recipients to prevent graft rejection. Calcineurin is released from its auto inhibitory loop upon T cell activation by antigen presentation and then dephosphorylates nuclear factor of activated T cells (NFAT) (53). NFAT migrates to the nucleus where it is responsible for the transcription of many pro-inflammatory genes, e.g. IL-2 (53). Besides NFAT, calcineurin inhibitors also target the mitogen-activated protein kinase (MAPK) pathway, and both these pathways play an important role in the myeloid cell lineage (53). The immunosuppressive properties of CsA could be linked to MDSC recruitment *in vitro* and *in vivo* (53). Daily treatment with CsA was found to increase MDSCs number, IDO and CXCR2 expression in a murine skin allograft transplantation model (53). Wang et al. suggested that the MDSC regulating effect of CsA is achieved by downregulation of NFATc1 (53). In addition, in skin grafted mice, the combined administration of GM-CSF and CsA was shown to increase MDSCs number and activity, by means of promoting iNOS expression (54). Therefore, calcineurin and NFAT appear to be interesting targets for MDSC regulation and induction.

#### 2.2.15 Glucosamine

Glucosamine is an essential substrate for the glycosylation of proteins and lipids. Recently, glucosamine was shown to promote the generation of MDSCs from murine bone marrow cells *in vitro* as well as in mice treated for 14 days with intraperitoneal injections of glucosamine (58). Furthermore, glucosamine also increased MDSC activity confirmed by T cell suppression assays and increased levels of Arg-1 and iNOS expression (58). Further analysis showed this effect was likely mediated *via* the STAT3 and ERK1/2 pathways (58).

#### 2.2.16 Glatirameracetat

GA has been found capable of promoting Tregs and Th2 lineage, while suppressing CD8^+^ T cell activity, and is therefore frequently used in relapsing-remitting MS (113). Recently, treatment with GA was demonstrated to increase the number and activity of MDSCs in a murine model of inflammatory bowel disease with improving health condition (66). The underlying mechanism was shown to involve binding of GA to the paired immunoglobulin (Ig) -like receptor B (PIR-B) in mice, or to the human ortholog leukocyte immunoglobulin-like receptor-B (LILRB) in humans (66). This interaction inhibited the STAT1 pathway and resulted in an increased IL-10 and TGF-β secretion (66). The results of inhibiting LILRBs in mice tumor models support this finding, since a decrease in MDSC number and STAT6 phosphorylation, a shift of the macrophage balance towards M2-like macrophages as well as increased STAT1/JAK activity had been observed, which all oppose MDSC promotion (67). As the regulation of the myeloid lineage is a highly complex net of signals in which the role of LILRBs is not fully explained yet, further research is needed.

### 2.3 Potential pharmacological targets on MDSCs in cancer

MDSCs accumulate in cancer and promote invasion, angiogenesis, metastasis formation and reduce the effectiveness of anti-tumor immunity (1, 3). Considering the negative role of MDSCs in the tumor microenvironment, one of the main goals of cancer research has been to suppress both MDSCs number and activity (1, 3). Many pharmacological targets have already been identified, which were shown to be involved in modulating the number and activity of MDSCs in different types of cancer, and were able to be targeted with different compounds (**Table 2**). As this review focusses on promoting MDSCs number and activity instead of inhibiting, in order to promote the anti-inflammatory immunity, the suggested MDSC-suppressing drugs are of course not of interest. However, the same targets that are used to suppress MDSCs in the context of the tumor microenvironment, might also be targeted in the complete opposite direction, in order to promote MDSCs in the context of potentially beneficial anti-inflammatory immunity. Several pharmacological targets that were manipulated to reduce the number and/or activity of MDSCs in the context of cancer, such as TLR4, β_2_-AR, EP4 and CXCR2, have already been described as potential targets for promoting the number and/or activity of MDSCs, as discussed above. For example, a β2-AR blocker, propranolol, was shown to reduce MDSC number and activity (20), while a β2-AR agonist, terbutaline, was shown to have the opposite effect (52). Similarly, the EP4 receptor antagonists, E7046 and YY001, were shown to reduce MDSC number and activity in the context of cancer (181, 182), while a EP4 receptor agonist, L-902,688, was shown to have the opposite effect in a murine model of asthma (57). However, there remain pharmacological targets of MDSC inhibition, which were identified in cancer research, that have so far not been studied in the context of MDSC promotion yet may provide efficient targets in novel anti-inflammatory therapies through generation and activation of these immune suppressive cells.

**Table 2:** Potential pharmacological targets on MDSCs in cancer.

Target	Potential pharmacological drug for cancer treatment	Effect of potential drug on MDSCs	Cancer type	Reference
A20	A20-siRNA	Reduced number	Lymphoma Melanoma	(114)
AMPKα	AMPKα inhibitor (dorsomorphin-compound), metformin	Reduced number Reduced activity	Colon carcinoma Esophageal carcinoma Lung carcinoma Ovarian carcinoma	(115–118)
Arginase-1, L-Arginine	L-Arginine, Arginase inhibitor (Nor-NOHA, CB-1158), ODC inhibitor (DFMO)	Reduced number Reduced activity	Colon carcinoma Melanoma Mammary carcinoma Ovarian carcinoma	(119–123)
Aurora A	Aurora A inhibitor (alisertib)	Reduced number	Mammary carcinoma	(124)
Bcl-xL	Bcl-xL inhibitor (ABT-737)	Reduced number	Colon carcinoma Mammary carcinoma	(125)
β2-AR/β3-AR	β-AR-blocker (propranolol), β3-AR-blocker (SR59230A)	Reduced number Reduced activity	Colon carcinoma Fibrosarcoma Mammary carcinoma Melanoma	(20, 126–128)
Caspases	Ganoderic acid A, caspase 8 inhibitor (Z-IETD-FMK)	Reduced number	Lung carcinoma Lymphoma	(129, 130)
CCL2/CCR2	CCR2 inhibitor (RS-102895, RS-504393), CCL2 inhibitor (BHC, propagermanium)	Reduced number	Basal cell carcinoma Bladder carcinoma Lung carcinoma Mammary carcinoma Rhabdomyosarcoma	(131–134)
CCL5/CCR5	CCL5 Ab, CCR5 inhibitor (Met-RANTES, Maraviroc)	Reduced number	Lymphoma Malignant melanoma Mammary carcinoma	(135–138)
CCRK	CCRK inhibitor	Reduced number	HCC	(139)
CD33	CD33 Ab (BI 836858), CD33/CD3-bispecific T-cell engager (AMG 330, AMV564)	Reduced number	Leukemia Melanoma	(140–142)
CD40	CD40 Ab	Reduced number Reduced activity	Colon carcinoma Gastric carcinoma Renal carcinoma	(143–146)
c-Rel (member of NF-κB family)	c-Rel inhibitor (R96A)	Reduced number	Lymphoma Melanoma	(147)
CSF-1/CSF-1 receptor	CSF-1 receptor inhibitor (GW2580, pexidartinib, PLX647, PLX5622, BLZ945)	Reduced number	Melanoma Neuroblastoma Prostate carcinoma	(148–151)
CXCR1/2/CXCL1/2/5	CXCR1/CXCR2 inhibitor (SX-682), CXCR2 inhibitor (SB-265610, benzocyclic sulfone derivatives) CXCR2 Ab	Reduced number	Colon carcinoma Mammary carcinoma Rhabdomyosarcoma	(4, 41, 152–158)
CXCR4	CXCR4 inhibitor (TFF2, plerixafor), poly polymerase inhibitor (olaparib), NAMPT inhibitor (FK866, MV87)	Reduced number	Colorectal carcinoma Fibrosarcoma Leukemia Mammary carcinoma Pancreatic carcinoma	(159–164)
D1(-like) receptor	Dopamine, D1-like receptor agonist (SKF38393)	Reduced activity	HNSCC Lung carcinoma Melanoma Prostate carcinoma	(165)
D2 receptor	D2 receptor agonist (cabergoline)	Reduced number	Lung carcinoma	(166)
Dectin-1	Dectin-1 agonist (WGP)	Reduced number Reduced activity	Lymphoma	(167)
Dkk1/β-catenin	Dkk1 Ab, galactosaminyltransferase	Reduced number	Lung carcinoma Melanoma	(168, 169)
DNA synthesis	Cytostatic drugs (gemcitabine, cisplatin, capecitabine, 5FU, lurbinectedin, 6-thioguanine, decitabine)	Reduced number	Bladder carcinoma Lymphoma Mammary carcinoma Myeloma Pancreatic carcinoma Thymoma	(170–179)
ENTPD2	ENTPD2 inhibitor	Reduced number	HCC	(180)
EP4 receptor	EP4 receptor antagonist (E7046, YY001)	Reduced number Reduced activity	Adenocarcinoma Colon carcinoma Mammary carcinoma Prostate carcinoma	(181, 182)
FAO	FAO inhibitor	Reduced activity	Colon carcinoma Lung carcinoma	(183)
Fcγ receptor	Fc portion of monoclonal Ab (Cetuximab)	Reduced activity	HNSCC	(184)
FGL2	FGL2 Ab	Reduced number	Glioma	(185)
G-CSF	G-CSF Ab, polyacetylenic glycoside (BP-E-F1)	Reduced number	Colorectal carcinoma Mammary carcinoma Sarcoma	(18, 186, 187)
Glutaminase/Glutathione/glutathione synthase	ATRA, glutaminase antagonist (JHU083)	Reduced number	Colorectal carcinoma Lung carcinoma Melanoma Mammary carcinoma Sarcoma	(188–195)
GM-CSF	GM-CSF Ab	Reduced number	HCC Mammary carcinoma Pancreatic carcinoma	(152, 196–198)
HDAC	HDAC inhibitor (entinostat, valproic acid)	Reduced number	Lung carcinoma Mammary carcinoma Melanoma Renal cell carcinoma	(199–201)
Histamine	Histamine antagonist (ranitidine), HDC	Reduced number Reduced activity	Colorectal carcinoma Lymphoma Mammary carcinoma	(202, 203)
HMGB1	HMGB1 inhibitor (ethyl pyruvate, glycyrrhizin)	Reduced number	Colon carcinoma Mammary carcinoma Melanoma	(204)
HOXA1	HOTAIRM1	Reduced activity	Lung carcinoma	(205)
IDO	IDO inhibitor (1-MT, INCB023843, EOS200271), IDO-vaccine, gemcitabine, superoxide dismutase mimetic	Reduced number Reduced activity	Colorectal carcinoma Lung carcinoma Mammary carcinoma Melanoma Pancreatic carcinoma	(46, 206–212)
IFN-γ	IFN-γ Ab	Reduced number	Colon carcinoma Leukemia Lymphoma Melanoma	(213)
IL-1β	IL-1 receptor antagonist, anti-IL-1β Ab	Reduced number	Gastric carcinoma Melanoma Prostate carcinoma	(27, 214, 215)
IL-4	IL-4 receptor-α blockade with RNA aptamer	Reduced activity	Fibrosarcoma Mammary carcinoma	(216, 217)
IL-6	IL-6-neutralizing Ab/IL-6-silencing vector	Reduced number	HCC Lung carcinoma Malignant melanoma Prostate carcinoma	(218–223)
IL-8	IL-8 Ab (HuMax-IL8)	Reduced number	Mammary carcinoma	(224, 225)
IL-10	IL-10 Ab, IL-10 receptor Ab	Reduced number Reduced activity	Mammary carcinoma Ovarian carcinoma	(226, 227)
IL-12	IL-12	Reduced number Reduced activity	Colon carcinoma Mammary carcinoma	(228, 229)
IL-13Rα2	IL-13-PE (immunotoxin of IL-13 fused to the Pseudomonas exotoxin A)	Reduced number	HNSCC	(230)
IL-18	IL-18 Ab (SK113AE4)	Reduced number	Melanoma Multiple myeloma Osteosarcoma	(231–233)
IL-33	IL-33 Ab	Reduced number Reduced activity	Melanoma	(234)
iNOS	iNOS inhibitor (L-NIL, L-NAME)	Reduced number	Colon carcinoma Lung carcinoma Lymphoma Malignant melanoma Melanoma Thymoma	(51, 235, 236)
IRF-4	IL4	Reduced number Reduced activity	Mammary carcinoma Melanoma	(237–239)
Jagged1/2	Anti-Jagged1/2-blocking Ab (CTX014)	Reduced number Reduced activity	Colon carcinoma Lung carcinoma Melanoma Thymoma	(240)
Kinases	Multikinase inhibitor (Sorafenib, Cabozantinib, BEZ235, Lenvatinib)	Reduced number Reduced activity	HCC Malignant melanoma Renal cell carcinoma Urothelial carcinoma	(197, 241)
Lactate	Anti-LDH Ab, ketogenic diet for glucose depletion	Reduced number	Pancreatic carcinoma	(242)
LILRB	LILRB antagonist	Reduced number	Lung carcinoma	30352428
LXR	LXR agonist (RGX-104, GW3965)	Reduced number	Colorectal carcinoma Lung carcinoma Melanoma Renal carcinoma Sarcoma Uterine carcinoma	(243, 244)
MEK/BRAF	MEK inhibitor (trametinib, cobimetinib, GDC-0623), BRAF inhibitor (Vemurafenib)	Reduced number	Colon carcinoma Lung carcinoma Mammary carcinoma Melanoma	(245–247)
MIF	MIF inhibitor (Sulforaphane)	Reduced number	Colon carcinoma Mammary carcinoma	(248)
mTOR	mTOR inhibitor (rapamycin, AZD2014, OSU-53)	Reduced number Reduced activity	Lung carcinoma Mammary carcinoma Melanoma Ovarian carcinoma	(249–252)
Myd88	Myd88 inhibitor (IMG2005, TJ-M2010-5)	Reduced number Reduced activity	Colorectal carcinoma	(253, 254)
NK1 receptor	Substance P	Reduced number	Mammary carcinoma	(255)
NOX2	NOX2 inhibitor	Reduced activity	Colon carcinoma Lung carcinoma Mammary carcinoma Sarcoma Thymoma	(44)
Osteactivin (DC-HIL)	Anti-DC-HIL Ab	Reduced activity	Colorectal carcinoma	(256)
P38 kinase	P38 inhibitor (LY2228820)	Reduced number	Lung carcinoma Mammary carcinoma Melanoma	(257, 258)
PD-L1	Anti-PD-L1 Ab	Reduced number	Colon carcinoma HNSCC Lung carcinoma Mammary carcinoma Melanoma Ovarian carcinoma	(259–262)
PI3K	PI3K inhibitor (IPI-145, Alpelisib, quinic acid), Artemisinin	Reduced number	Colon carcinoma HNSCC Mammary carcinoma Melanoma	(263–266)
PPARγ	PPARy agonists	Reduced number Reduced activity	Lung carcinoma	(267, 268)
PNT	PNT inhibitor (CDDO-Me), PNT scavenger (MnTBAP)	Reduced number Reduced activity	Lung carcinoma Melanoma Thymoma	(51, 269)
Phosphatidylserine	Anti-phosphatidylserine Ab (Bavituximab)	Reduced number	Mammary carcinoma Prostate carcinoma	(270, 271)
PDE-5	PDE-5 inhibitor (Sildenafil, Tadalafil), Paclitaxel	Reduced number Reduced activity	Colon carcinoma Lymphoma Melanoma	(235, 272–277)
PGE_2_	Celecoxib, SC58125, SC58236 (cyclooxygenase 2 inhibitors), Indomethancin (IND), EP2/4 antagonist	Reduced number Reduced activity	Colon carcinoma Lung carcinoma Mammary carcinoma Melanoma Ovarian carcinoma Pancreatic carcinoma	(41, 55, 278–283)
Rac	Rac inhibitor (EHT-1864)	Reduced number	Colitis-associated carcinoma	(284)
RLH	RLH ligand (polyinosinic-polycytidylic acid (poly(I:C))	Reduced activity	Pancreatic carcinoma	31694706
RORC1/RORγ	RORC1 inhibitor	Reduced number	Sarcoma	(285)
ROS	CDDO-Me (Triterpenoid), Doxorubicin	Reduced activity	Colon carcinoma Lung carcinoma Mammary carcinoma Renal cell carcinoma Sarcoma Thymoma	(286, 287)
RTKs/BTK	RTK inhibitor (Sunitinib, nilotinib, dasatinib, sorafenib, UNC4241), BTK inhibitor (ibrutinib)	Reduced number Reduced activity	Cervical carcinoma Colon carcinoma HNSCC Mammary carcinoma Melanoma Pancreatic carcinoma Renal cell carcinoma	(288–301)
S100A8/9/RAGE	anti-S100A8/9 Ab, S100A9 inhibitor (Tasquinimod), Anti-RAGE Ab	Reduced number Reduced activity	Colon carcinoma Gastric carcinoma Lung carcinoma Lymphoma Mammary carcinoma Sarcoma	(189, 302–309)
S1P	LCL521	Reduced activity	HNSCC Sarcoma	(310, 311)
SCARB1	Synthethic high-density lipoprotein-like nanoparticles	Reduced activity	Melanoma	(312)
Semaphorin 4D	Semaphorin 4D Ab	Reduced number Reduced activity	HNSCC	(313, 314)
SIRT1 *via* HIF-1α	SIRT1 activator (SRT1720), HIF-1α inhibitor (2-ME)	Reduced number	Lymphoma Melanoma	(315)
STAT3	STAT3 inhibitor (JSI-124, Stattic, AG490, Nifuroxazide, S3I, FLLL32, BBI608, napabucasin, quercetin), Sunitinib (TKI), Curcumin, Notch signaling blocker (selective CK2 inhibitor (TBCA), (γ-secretase inhibitor (GSI-IX, DAPT))	Reduced number Reduced activity	Colon carcinoma Gastric carcinoma HCC HNSCC Lung carcinoma Lymphoma Mammary carcinoma Melanoma Ovarian carcinoma Pancreatic carcinoma Renal cell carcinoma Sarcoma	(23, 32, 46, 60, 316–331)
STING	STING agonist	Reduced number	Colorectal carcinoma Nasopharyngeal carcinoma	(211, 332)
TGF-β1	Anti-TGF-β1 Ab, TGF-β inhibitor (Pirfenidone)	Reduced number Reduced activity	HCC Lung carcinoma Lymphoma Melanoma Renal cell carcinoma	(309, 333)
TLR1/2	TLR1/TLR2 agonist (synthethic bacterial lipoprotein), HSP70 ligand/blocker (A8 peptide)	Reduced number	Lung carcinoma Lymphoma Melanoma	(334, 335)
TLR4	TLR4-inducer (Asparagus polysaccharide, cinnamaldehyde)	Reduced number Reduced activity	Colon carcinoma Colorectal carcinoma	(336, 337)
TLR7/8	TLR7 agonist (imiqimod), TLR8 agonist (motolimod), TLR7/8 agonist (resiquimod)	Reduced number Reduced activity	HNSCC Melanoma	(338–342)
TLR9	TLR9 agonist (CpG), TLR9-targeted STAT3siRNA	Reduced number Reduced activity	Colon carcinoma Gastric carcinoma Prostatic carcinoma	(343, 344)
TNF/TNFR2	TNF Ab/inhibitor (XPro1595, etanercept, infliximab), TNFR2 inhibitor (TNFR2 antisense oligodeoxynucleotides, TNFR2-Fc fusion protein)	Reduced number	Colon carcinoma Lung carcinoma Sarcoma	(345, 346)
TRAIL receptors	TRAIL receptor 2 agonist (DS-8273a)	Reduced number	Advanced stage solid tumors Lung carcinoma	(347, 348)
Nrf2	Nrf2 inducer (CDDO-Im)	Reduced activity	Lung carcinoma	(349)
VEGF/VEGF receptor	VEGF receptor inhibitor (SAR131675, pazopanib), VEGF Ab (bevacizumab)	Reduced number	Lung carcinoma Mammary carcinoma Prostate carcinoma	(350–354)

1-MT, 1-methyl-L-tryptophan; 2-ME, 2-Methoxyestradiol; 5FU, 5-fluorouracil; Ab, Antibody; AR, Adrenergicreceptor; AMPKα, AMP-activated protein kinase alpha; ATRA, All-trans retinoic acid; Bcl-xL, B-cell lymphoma-extra large; BHC, Benzamide hydrochloride; BTK, Bruton’s tyrosine kinase; BRAF, v-RAF murine sarcoma viral oncogene homolog B gene; CCL, Chemokine (C-C motif) ligand; CCR, C-C chemokine receptor; CCRK, Cell cycle-related kinase; CD, Cluster of differentiation; CDDO-Im, CDDO-Imidazolide; CDDO-Me, Methyl-2-cyano-3,12-dioxooleana-1,9 (11)-dien-28-oate; CK2, Casein kinase 2; CSF-1, Colony stimulating factor-1; CXCL, Chemokine (C-X-C motif) ligand; CXCR, C-X-C chemokine receptor; DC-HIL, Dendritic cell heparan sulfate proteoglycan integrin-dependent ligand; DFMO, Difluoromethylornithine; Dkk1, Dickkopf-related protein 1; ENTPD2, Ectonucleoside triphosphate diphosphohydrolase 2; ER, Endoplasmic reticulum; FAO, Fatty acid oxidation; FGL2, Fibrinogen-like protein 2; G-CSF, Granulocyte-colony stimulating factor; GM-CSF, Granulocyte-macrophage colony stimulating factor; HCC, Hepatocellular carcinoma; HDAC, Histone deacetylase; HDC, Histamine dihydrochloride; HIF-1α, Hypoxia-inducible factor 1α; HMGB1, High-Mobility-Group Protein B1; HNSCC, Head and neck squamous cell carcinoma; HOXA1, Homeobox protein Hox-A1; HOTAIRM1, HOXA transcript antisense RNA myeloid specific 1; IDO, Indoleamine-pyrrole 2,3-dioxygenase; IFN, Interferon; IL, Interleukin; iNOS, Inducable nitric oxide synthases; IRF-4, Interferon-regulatory factor 4; LDH, Lactate dehydrogenase; LILRB, Leukocyte immunoglobulin-like receptor B; L-NAME, N(G) nitro L-arginine methyl ester; L-NIL, N6-(1-Iminoethyl)-lysine; LXR, Liver-X nuclear receptor; MEK, Mitogen-activated protein kinase kinase; MIF, Macrophage migration inhibitory factor; MnTBAP, Mn(III)tetrakis(4-benzoic acid)porphyrin; mTOR, Mammalian target of rapamycin; Myd88, Myeloid differentiation primary response 88; NAMPT, Nicotinamide phosphoribosyltransferase; NAPDH, Nicotinamide adenine dinucleotide phosphate; NK1, Neurokinin 1; Nor-NOHA, Nω-Hydroxy-nor-L-arginine; NOX2, NADPH oxidase 2; ODC, Ornithine decarboxylase; Nrf2, Transcription factor NF-E2-related factor-2; PDE-5, Phosphodiesterase-5; PD-L1, Programmed death-ligand 1; PGE2, Prostaglandin E2; PI3K, Phosphoinositide 3-kinase; PNT, Peroxynitrite; PPARγ, Peroxisome proliferator-activated receptor-γ; RAGE, Receptor for advanced glycation endproducts; RANTES, Regulated upon Activation, Normal T cell Expressed and presumably Secreted; RLH, RIG-I-like helicase; ROS, Reactive oxygen species; siRNA, Small interfering RNA; RORC1/RORγ, Retinoic-acid-related orphan receptor; SCARB1, Scavenger receptor type B-1; SIRT1, Sirtuin (silent mating type information regulation 2 homolog) 1; STAT3, Signal transducer and activator of transcription 3; STING, Stimulator of interferon genes; TBCA, Tetrabromocinnamic acid [(E)-3-(2,3,4,5-tetrabromophenyl)acrylic acid; TGF-β, Transforming growth factor β; TNF, Tumor necrose factor; WGP, Whole β-glucan particles; RTK, Receptor tyrosine kinase; TLR, Toll-like receptor; VEGFR, Vascular endothelial growth factor.

## 3 Discussion

Findings on the modulation of the generation, recruitment, and activation of MDSCs open the door to novel pharmacological approaches that can be used to dampen inflammation in a variety of diseases or conditions characterized by excessive and detrimental immune responses (such as autoimmune diseases, chronic inflammatory diseases, transplantation and GVHD). Taking all the mentioned mechanisms and the potential pharmacological targets into consideration, the *in vitro* generation of MDSCs combined with adoptive transfer (MDSC cell therapy) as well as the *in vivo* recruitment and activation of endogenous MDSCs seem to both be promising approaches. Nonetheless, cell therapies always bear additional risks, e.g. reaction of the immune system against transferred cells, and adverse side effects, and, as a result, need to be studied carefully before applying it to the clinical setting. Alternatively, the promotion of endogenous MDSCs could create safer, easier, and potentially equally or more efficient MDSC-targeted therapies in the future.

On the one hand, *in vitro* generation of MDSCs with the help of cytokines – like GM-CSF and IL-6 – or pharmacological compounds – such as PGE_2_ –, followed by adoptive transfer is an effective and increasingly established treatment in murine models of chronic inflammatory diseases, autoimmune diseases, and allograft transplantation. Here, it is possible to circumvent the potential systemic side effects of unspecific pharmacological compounds and transfer a purified subset of immunosuppressive cells to dampen inflammation. On the other hand, efficient endogenous MDSC generation and activation was achieved with a double CFA injection containing *M. tuberculosis* or MVs through the TLR pathway, mTOR inhibitor rapamycin, dexamethasone, GA, EP4 receptor agonist as well as with other medications (48, 56, 57, 63, 64, 66, 67, 79, 81, 82, 84, 86, 87). Furthermore, many chemokines, and their receptors, have been identified to be involved in MDSC recruitment, with the most noteworthy chemokine being CXCR2, with its most important ligands CXCL1 and CXCL2. CXCR2 ligands can be used to regulate the migration of generated MDSCs to the site of interest, e.g. the intestines in Crohn’s disease, or the lungs of asthmatic patients. In this context, allografts could be coated with chemokine agonists to promote local MDSC accumulation - similar to vascular stents coated with anticoagulants and anti-proliferative drugs, like rapamycin. However, the *in vivo* generation of endogenous MDSCs may be a more straightforward therapy compared to MDSC cell therapy, which would ideally be accomplished with a selective drug and pharmacological targets, in order to reduce possible side effects as much as possible.

The safety of adoptively transferred MDSCs is not very well known and remains one of the primary challenges in bringing MDSC therapy to a clinical setting (355). Similarly, the safety of inducing endogenous MDSCs remains largely unknown. One concern may be the immature characteristic of MDSCs, which makes them susceptible to the induction of differentiation into other immune cells, such as macrophages, neutrophils or DCs, depending on specific microenvironmental cues. The negative role of MDSCs in the context of cancer also raises the following questions: Can MDSC inducing therapy only be applied in patients who are “certainly tumor-free”? And if so, how is it possible to identify this patient group? Or is the expected beneficial effect so great that the increased risk of metastasis can be accepted? Is there a way to inhibit MDSCs in undesired parts of the body? As the vast majority of studies on MDSCs are cancer-related and the systemic MDSC induction has been linked to tumor progression, metastasis and impaired survival, a method of local induction of MDSCs would be necessary to reduce the risk of MDSC-induced cancer progression as well as potential other side effects of systemic immunosuppression, e.g. opportunistic infections.

The increasing number of potential pharmacological targets that have been shown to be involved in modulating the number and activity of MDSCs provide significant number of opportunities for novel pharmacological approaches. Especially considering the potential targets, which were previously mainly studied in the context of cancer (**Table 2**), to inhibit the MDSC response, where this effect may be reversed (e.g. changing from an agonist to antagonist or the other way around), in order to promote the MDSC response. The interest in the modulation of MDSC outside the context of cancer is also increasing, as the importance of MDSCs in many different immune-related pathological conditions is being unraveled (356). Furthermore, transplantation research has also increased its attention towards MDSC immunomodulation as a promising candidate to increase tolerance and improve transplant outcome (357). Thus, the pharmacological approaches which can be applied in MDSC modulation, as discussed in this review, may provide novel opportunities in the future of tolerance-inducing agents in the context of transplantation.

Taken together, there are many different promising pharmacological targets to generate and activate MDSCs, and their beneficial potential in certain pathological conditions has been established in animal studies. Therefore, MDSC therapies may prove to be effective alternatives to other immunosuppressive therapies. The selective harnessing of regulatory immune cells, here expanding suppressive activity of MDSCs on T cells, may bring an advanced possibility to protect, limit or ameliorate the initiation or progression of autoimmunity, inflammation, transplant rejection or GVHD, promote inflammation resolution and transplantation tolerance and pave the way toward precise medical therapy in inflammation/autoimmunity/transplant medicine.

## Author contributions

SK conceptualized the review. CG, CH, AD and SK contributed to the original draft. CG and SK contributed to revising and final approval of the manuscript. All authors contributed to the article and approved the submitted version.

## Funding

This study was funded by the Universities Giessen Marburg Lung Center (UGMLC) and the German Center for Lung Disease (DZL German Lung Center, no. 82DZL005B2), the Foundation for Pathobiochemistry and Molecular Diagnostics grant and the ƒortüne program of the University of Tuebingen (# 2458-0-0, # 2606-0-0) for SK. Open access was funded by the Open Access Publishing Fund of Philipps-Universität Marburg with support of the Deutsche Forschungsgemeinschaft (DFG, German Research Foundation) and the Open Access Publishing Fund of the University of Tübingen.

## Conflict of interest

The authors declare that the research was conducted in the absence of any commercial or financial relationships that could be construed as a potential conflict of interest.

## Publisher’s note

All claims expressed in this article are solely those of the authors and do not necessarily represent those of their affiliated organizations, or those of the publisher, the editors and the reviewers. Any product that may be evaluated in this article, or claim that may be made by its manufacturer, is not guaranteed or endorsed by the publisher.

## Footnotes

1
ClinicalTrials.gov

2
Clinicaltrialsregister.eu

## References

[1] Gabrilovich DI Nagaraj S. Myeloid-derived suppressor cells as regulators of the immune system. Nat Rev Immunol (2009) 9(3):162–74. doi: 10.1038/nri2506 PMC2828349 19197294

[2] Bronte V Apolloni E Cabrelle A Ronca R Serafini P Zamboni P. Identification of a Cd11b(+)/Gr-1(+)/Cd31(+) myeloid progenitor capable of activating or suppressing Cd8(+) T cells. Blood (2000) 96(12):3838–46. doi: 10.1182/blood.V96.12.3838 PMC2734459 11090068

[3] Talmadge JE Gabrilovich DI. History of myeloid-derived suppressor cells. Nat Rev Cancer (2013) 13(10):739–52. doi: 10.1038/nrc3581 PMC4358792 24060865

[4] Sun L Clavijo PE Robbins Y Patel P Friedman J Greene S. Inhibiting myeloid-derived suppressor cell trafficking enhances T cell immunotherapy. JCI Insight (2019) 4(7):e126853. doi: 10.1172/jci.insight.126853 PMC6483637 30944253

[5] Albeituni SH Ding C Yan J. Hampering immune suppressors: Therapeutic targeting of myeloid-derived suppressor cells in cancer. Cancer J (2013) 19(6):490–501. doi: 10.1097/PPO.0000000000000006 24270348 PMC3902636

[6] Dorhoi A Du Plessis N. Monocytic myeloid-derived suppressor cells in chronic infections. Front Immunol (2017) 8:1895. doi: 10.3389/fimmu.2017.01895 29354120 PMC5758551

[7] Cripps JG Gorham JD. Mdsc in autoimmunity. Int Immunopharmacol (2011) 11(7):789–93. doi: 10.1016/j.intimp.2011.01.026 PMC3109222 21310255

[8] Deshane JS Redden DT Zeng M Spell ML Zmijewski JW Anderson JT. Subsets of airway myeloid-derived regulatory cells distinguish mild asthma from chronic obstructive pulmonary disease. J Allergy Clin Immunol (2015) 135(2):413–24 e15. doi: 10.1016/j.jaci.2014.08.040 25420684 PMC4323991

[9] Clements VK Long T Long R Figley C Smith DMC Ostrand-Rosenberg S. Frontline science: High fat diet and leptin promote tumor progression by inducing myeloid-derived suppressor cells. J Leukoc Biol (2018) 103(3):395–407. doi: 10.1002/JLB.4HI0517-210R 29345342 PMC7414791

[10] Pawelec G Verschoor CP Ostrand-Rosenberg S. Myeloid-derived suppressor cells: Not only in tumor immunity. Front Immunol (2019) 10:1099. doi: 10.3389/fimmu.2019.01099 31156644 PMC6529572

[11] Bronte V Brandau S Chen SH Colombo MP Frey AB Greten TF. Recommendations for myeloid-derived suppressor cell nomenclature and characterization standards. Nat Commun (2016) 7:12150. doi: 10.1038/ncomms12150 27381735 PMC4935811

[12] Condamine T Dominguez GA Youn JI Kossenkov AV Mony S Alicea-Torres K. Lectin-type oxidized ldl receptor-1 distinguishes population of human polymorphonuclear myeloid-derived suppressor cells in cancer patients. Sci Immunol (2016) 1(2):aaf8943. doi: 10.1126/sciimmunol.aaf8943 28417112 PMC5391495

[13] Alshetaiwi H Pervolarakis N McIntyre LL Ma D Nguyen Q Rath JA. Defining the emergence of myeloid-derived suppressor cells in breast cancer using single-cell transcriptomics. Sci Immunol (2020) 5(44):eaay6017. doi: 10.1126/sciimmunol.aay6017 32086381 PMC7219211

[14] Katzenelenbogen Y Sheban F Yalin A Yofe I Svetlichnyy D Jaitin DA. Coupled scrna-seq and intracellular protein activity reveal an immunosuppressive role of Trem2 in cancer. Cell (2020) 182(4):872–85 e19. doi: 10.1016/j.cell.2020.06.032 32783915

[15] Condamine T Mastio J Gabrilovich DI. Transcriptional regulation of myeloid-derived suppressor cells. J Leukoc Biol (2015) 98(6):913–22. doi: 10.1189/jlb.4RI0515-204R PMC4661041 26337512

[16] Thorn M Guha P Cunetta M Espat NJ Miller G Junghans RP. Tumor-associated gm-csf overexpression induces immunoinhibitory molecules *Via* Stat3 in myeloid-suppressor cells infiltrating liver metastases. Cancer Gene Ther (2016) 23(6):188–98. doi: 10.1038/cgt.2016.19 27199222

[17] Peng D Tanikawa T Li W Zhao L Vatan L Szeliga W. Myeloid-derived suppressor cells endow stem-like qualities to breast cancer cells through Il6/Stat3 and No/Notch cross-talk signaling. Cancer Res (2016) 76(11):3156–65. doi: 10.1158/0008-5472.CAN-15-2528 PMC4891237 27197152

[18] Li W Zhang X Chen Y Xie Y Liu J Feng Q. G-Csf is a key modulator of mdsc and could be a potential therapeutic target in colitis-associated colorectal cancers. Protein Cell (2016) 7(2):130–40. doi: 10.1007/s13238-015-0237-2 PMC4742385 26797765

[19] Waight JD Netherby C Hensen ML Miller A Hu Q Liu S. Myeloid-derived suppressor cell development is regulated by a Stat/Irf-8 axis. J Clin Invest (2013) 123(10):4464–78. doi: 10.1172/JCI68189 PMC3784535 24091328

[20] Mohammadpour H MacDonald CR Qiao G Chen M Dong B Hylander BL. Beta2 adrenergic receptor-mediated signaling regulates the immunosuppressive potential of myeloid-derived suppressor cells. J Clin Invest (2019) 129(12):5537–52. doi: 10.1172/JCI129502 PMC6877316 31566578

[21] Moreira D Adamus T Zhao X Su YL Zhang Z White SV. Stat3 inhibition combined with cpg immunostimulation activates antitumor immunity to eradicate genetically distinct castration-resistant prostate cancers. Clin Cancer Res (2018) 24(23):5948–62. doi: 10.1158/1078-0432.CCR-18-1277 PMC6279477 30337279

[22] Hellsten R Lilljebjorn L Johansson M Leandersson K Bjartell A. The Stat3 inhibitor galiellalactone inhibits the generation of mdsc-like monocytes by prostate cancer cells and decreases immunosuppressive and tumorigenic factors. Prostate (2019) 79(14):1611–21. doi: 10.1002/pros.23885 PMC6771992 31348843

[23] Guha P Gardell J Darpolor J Cunetta M Lima M Miller G. Stat3 inhibition induces bax-dependent apoptosis in liver tumor myeloid-derived suppressor cells. Oncogene (2019) 38(4):533–48. doi: 10.1038/s41388-018-0449-z 30158673

[24] Mundy-Bosse BL Lesinski GB Jaime-Ramirez AC Benninger K Khan M Kuppusamy P. Myeloid-derived suppressor cell inhibition of the ifn response in tumor-bearing mice. Cancer Res (2011) 71(15):5101–10. doi: 10.1158/0008-5472.CAN-10-2670 PMC3148319 21680779

[25] Schroder M Krotschel M Conrad L Naumann SK Bachran C Rolfe A. Genetic screen in myeloid cells identifies tnf-alpha autocrine secretion as a factor increasing mdsc suppressive activity *Via* Nos2 up-regulation. Sci Rep (2018) 8(1):13399. doi: 10.1038/s41598-018-31674-1 30194424 PMC6128861

[26] Porta C Consonni FM Morlacchi S Sangaletti S Bleve A Totaro MG. Tumor-derived prostaglandin E2 promotes P50 nf-Kappab-Dependent differentiation of monocytic mdscs. Cancer Res (2020) 80(13):2874–88. doi: 10.1158/0008-5472.CAN-19-2843 32265223

[27] Tu S Bhagat G Cui G Takaishi S Kurt-Jones EA Rickman B. Overexpression of interleukin-1beta induces gastric inflammation and cancer and mobilizes myeloid-derived suppressor cells in mice. Cancer Cell (2008) 14(5):408–19. doi: 10.1016/j.ccr.2008.10.011 PMC2586894 18977329

[28] Hu CE Gan J Zhang RD Cheng YR Huang GJ. Up-regulated myeloid-derived suppressor cell contributes to hepatocellular carcinoma development by impairing dendritic cell function. Scand J Gastroenterol (2011) 46(2):156–64. doi: 10.3109/00365521.2010.516450 20822377

[29] Tannenbaum CS Rayman PA Pavicic PG Kim JS Wei W Polefko A. Mediators of inflammation-driven expansion, trafficking, and function of tumor-infiltrating mdscs. Cancer Immunol Res (2019) 7(10):1687–99. doi: 10.1158/2326-6066.CIR-18-0578 PMC6774821 31439615

[30] Choi JN Sun EG Cho SH. Il-12 enhances immune response by modulation of myeloid derived suppressor cells in tumor microenvironment. Chonnam Med J (2019) 55(1):31–9. doi: 10.4068/cmj.2019.55.1.31 PMC6351325 30740338

[31] Yan G Zhao H Zhang Q Zhou Y Wu L Lei J. A Ripk3-Pge2 circuit mediates myeloid-derived suppressor cell-potentiated colorectal carcinogenesis. Cancer Res (2018) 78(19):5586–99. doi: 10.1158/0008-5472.CAN-17-3962 30012671

[32] Cheng P Kumar V Liu H Youn JI Fishman M Sherman S. Effects of notch signaling on regulation of myeloid cell differentiation in cancer. Cancer Res (2014) 74(1):141–52. doi: 10.1158/0008-5472.CAN-13-1686 PMC3886562 24220241

[33] Morello S Miele L. Targeting the adenosine A2b receptor in the tumor microenvironment overcomes local immunosuppression by myeloid-derived suppressor cells. Oncoimmunology (2014) 3:e27989. doi: 10.4161/onci.27989 25101221 PMC4121336

[34] Tcyganov E Mastio J Chen E Gabrilovich DI. Plasticity of myeloid-derived suppressor cells in cancer. Curr Opin Immunol (2018) 51:76–82. doi: 10.1016/j.coi.2018.03.009 29547768 PMC5943174

[35] Li BH Garstka MA Li ZF. Chemokines and their receptors promoting the recruitment of myeloid-derived suppressor cells into the tumor. Mol Immunol (2020) 117:201–15. doi: 10.1016/j.molimm.2019.11.014 31835202

[36] Hsu YL Yen MC Chang WA Tsai PH Pan YC Liao SH. Cxcl17-derived Cd11b(+)Gr-1(+) myeloid-derived suppressor cells contribute to lung metastasis of breast cancer through platelet-derived growth factor-bb. Breast Cancer Res (2019) 21(1):23. doi: 10.1186/s13058-019-1114-3 30755260 PMC6373011

[37] Oliveira AC Fu C Lu Y Williams MA Pi L Brantly ML. Chemokine signaling axis between endothelial and myeloid cells regulates development of pulmonary hypertension associated with pulmonary fibrosis and hypoxia. Am J Physiol Lung Cell Mol Physiol (2019) 317(4):L434–L44. doi: 10.1152/ajplung.00156.2019 PMC6842914 31364370

[38] Taki M Abiko K Baba T Hamanishi J Yamaguchi K Murakami R. Snail promotes ovarian cancer progression by recruiting myeloid-derived suppressor cells *Via* Cxcr2 ligand upregulation. Nat Commun (2018) 9(1):1685. doi: 10.1038/s41467-018-03966-7 29703902 PMC5923228

[39] Neamah WH Singh NP Alghetaa H Abdulla OA Chatterjee S Busbee PB. Ahr activation leads to massive mobilization of myeloid-derived suppressor cells with immunosuppressive activity through regulation of Cxcr2 and microrna mir-150-5p and mir-543-3p that target anti-inflammatory genes. J Immunol (2019) 203(7):1830–44. doi: 10.4049/jimmunol.1900291 PMC6755129 31492743

[40] Zhang C Wang S Li J Zhang W Zheng L Yang C. The mtor signal regulates myeloid-derived suppressor cells differentiation and immunosuppressive function in acute kidney injury. Cell Death Dis (2017) 8(3):e2695. doi: 10.1038/cddis.2017.86 28333137 PMC5386577

[41] Katoh H Wang D Daikoku T Sun H Dey SK Dubois RN. Cxcr2-expressing myeloid-derived suppressor cells are essential to promote colitis-associated tumorigenesis. Cancer Cell (2013) 24(5):631–44. doi: 10.1016/j.ccr.2013.10.009 PMC3928012 24229710

[42] Han X Shi H Sun Y Shang C Luan T Wang D. Cxcr2 expression on granulocyte and macrophage progenitors under tumor conditions contributes to Mo-mdsc generation *Via* Sap18/Erk/Stat3. Cell Death Dis (2019) 10(8):598. doi: 10.1038/s41419-019-1837-1 31395859 PMC6687752

[43] Ohl K Tenbrock K. Reactive oxygen species as regulators of mdsc-mediated immune suppression. Front Immunol (2018) 9:2499. doi: 10.3389/fimmu.2018.02499 30425715 PMC6218613

[44] Corzo CA Cotter MJ Cheng P Cheng F Kusmartsev S Sotomayor E. Mechanism regulating reactive oxygen species in tumor-induced myeloid-derived suppressor cells. J Immunol (2009) 182(9):5693–701. doi: 10.4049/jimmunol.0900092 PMC2833019 19380816

[45] Rodriguez PC Quiceno DG Ochoa AC. L-arginine availability regulates T-lymphocyte cell-cycle progression. Blood (2007) 109(4):1568–73. doi: 10.1182/blood-2006-06-031856 PMC1794048 17023580

[46] Yu J Du W Yan F Wang Y Li H Cao S. Myeloid-derived suppressor cells suppress antitumor immune responses through ido expression and correlate with lymph node metastasis in patients with breast cancer. J Immunol (2013) 190(7):3783–97. doi: 10.4049/jimmunol.1201449 23440412

[47] Huang B Pan PY Li Q Sato AI Levy DE Bromberg J. Gr-1+Cd115+ immature myeloid suppressor cells mediate the development of tumor-induced T regulatory cells and T-cell anergy in tumor-bearing host. Cancer Res (2006) 66(2):1123–31. doi: 10.1158/0008-5472.CAN-05-1299 16424049

[48] Nakamura T Nakao T Yoshimura N Ashihara E. Rapamycin prolongs cardiac allograft survival in a mouse model by inducing myeloid-derived suppressor cells. Am J Transplant (2015) 15(9):2364–77. doi: 10.1111/ajt.13276 25943210

[49] Wang Y Schafer CC Hough KP Tousif S Duncan SR Kearney JF. Myeloid-derived suppressor cells impair b cell responses in lung cancer through il-7 and Stat5. J Immunol (2018) 201(1):278–95. doi: 10.4049/jimmunol.1701069 PMC6008229 29752311

[50] Youn JI Nagaraj S Collazo M Gabrilovich DI. Subsets of myeloid-derived suppressor cells in tumor-bearing mice. J Immunol (2008) 181(8):5791–802. doi: 10.4049/jimmunol.181.8.5791 PMC2575748 18832739

[51] Raber PL Thevenot P Sierra R Wyczechowska D Halle D Ramirez ME. Subpopulations of myeloid-derived suppressor cells impair T cell responses through independent nitric oxide-related pathways. Int J Cancer (2014) 134(12):2853–64. doi: 10.1002/ijc.28622 PMC3980009 24259296

[52] Mohammadpour H Sarow JL MacDonald CR Chen GL Qiu J Sharma UC. Beta2-adrenergic receptor activation on donor cells ameliorates acute gvhd. JCI Insight (2020) 5(12):e137788. doi: 10.1172/jci.insight.137788 PMC7406296 32437333

[53] Wang X Bi Y Xue L Liao J Chen X Lu Y. The calcineurin-nfat axis controls allograft immunity in myeloid-derived suppressor cells through reprogramming T cell differentiation. Mol Cell Biol (2015) 35(3):598–609. doi: 10.1128/MCB.01251-14 25452304 PMC4285420

[54] Han C Wu T Na N Zhao Y Li W Zhao Y. The effect of immunosuppressive drug cyclosporine a on myeloid-derived suppressor cells in transplanted mice. Inflamm Res (2016) 65(9):679–88. doi: 10.1007/s00011-016-0949-7 27147271

[55] Obermajer N Muthuswamy R Lesnock J Edwards RP Kalinski P. Positive feedback between Pge2 and Cox2 redirects the differentiation of human dendritic cells toward stable myeloid-derived suppressor cells. Blood (2011) 118(20):5498–505. doi: 10.1182/blood-2011-07-365825 PMC3217352 21972293

[56] Jontvedt Jorgensen M Jenum S Tonby K Mortensen R Walzl G Du Plessis N. Monocytic myeloid-derived suppressor cells reflect tuberculosis severity and are influenced by cyclooxygenase-2 inhibitors. J Leukoc Biol (2020) 110(1):177–86. doi: 10.1002/JLB.4A0720-409RR PMC8359170 33155730

[57] van Geffen C Deißler A Beer-Hammer S Nürnberg B Handgretinger R Renz H. Myeloid-derived suppressor cells dampen airway inflammation through prostaglandin E2 receptor 4. Front Immunol (2021) 12:695933. doi: 10.3389/fimmu.2021.695933 34322123 PMC8311661

[58] Lin EC Chen SW Chen LK Lin TA Wu YX Juan CC. Glucosamine interferes with myelopoiesis and enhances the immunosuppressive activity of myeloid-derived suppressor cells. Front Nutr (2021) 8:762363. doi: 10.3389/fnut.2021.762363 34901113 PMC8660085

[59] Vafadar A Shabaninejad Z Movahedpour A Fallahi F Taghavipour M Ghasemi Y. Quercetin and cancer: New insights into its therapeutic effects on ovarian cancer cells. Cell Biosci (2020) 10:32. doi: 10.1186/s13578-020-00397-0 32175075 PMC7063794

[60] Ma Z Xia Y Hu C Yu M Yi H. Quercetin promotes the survival of granulocytic myeloid-derived suppressor cells *via* the Esr2/Stat3 signaling pathway. BioMed Pharmacother (2020) 125:109922. doi: 10.1016/j.biopha.2020.109922 32007919

[61] Davenport AP Hyndman KA Dhaun N Southan C Kohan DE Pollock JS. Endothelin. Pharmacol Rev (2016) 68(2):357–418. doi: 10.1124/pr.115.011833 26956245 PMC4815360

[62] Chen Z Zhang X Lv S Xing Z Shi M Li X. Treatment with endothelin-a receptor antagonist Bq123 attenuates acute inflammation in mice through T-Cell-Dependent polymorphonuclear myeloid-derived suppressor cell activation. Front Immunol (2021) 12:641874. doi: 10.3389/fimmu.2021.641874 33828553 PMC8019801

[63] Zhao Y Shen XF Cao K Ding J Kang X Guan WX. Dexamethasone-induced myeloid-derived suppressor cells prolong allo cardiac graft survival through inos- and glucocorticoid receptor-dependent mechanism. Front Immunol (2018) 9:282. doi: 10.3389/fimmu.2018.00282 29497426 PMC5818399

[64] Liao J Wang X Bi Y Shen B Shao K Yang H. Dexamethasone potentiates myeloid-derived suppressor cell function in prolonging allograft survival through nitric oxide. J Leukoc Biol (2014) 96(5):675–84. doi: 10.1189/jlb.2HI1113-611RR 24948701

[65] Wang Z Zheng G Li G Wang M Ma Z Li H. Methylprednisolone alleviates multiple sclerosis by expanding myeloid-derived suppressor cells *via* glucocorticoid receptor beta and S100a8/9 up-regulation. J Cell Mol Med (2020) 24(23):13703–14. doi: 10.1111/jcmm.15928 PMC7753844 33094923

[66] van der Touw W Kang K Luan Y Ma G Mai S Qin L. Glatiramer acetate enhances myeloid-derived suppressor cell function *Via* recognition of paired ig-like receptor b. J Immunol (2018) 201(6):1727–34. doi: 10.4049/jimmunol.1701450 PMC6379207 30068593

[67] Chen HM van der Touw W Wang YS Kang K Mai S Zhang J. Blocking immunoinhibitory receptor Lilrb2 reprograms tumor-associated myeloid cells and promotes antitumor immunity. J Clin Invest (2018) 128(12):5647–62. doi: 10.1172/JCI97570 PMC6264729 30352428

[68] Liu Y Perego M Xiao Q He Y Fu S He J. Lactoferrin-induced myeloid-derived suppressor cell therapy attenuates pathologic inflammatory conditions in newborn mice. J Clin Invest (2019) 129(10):4261–75. doi: 10.1172/JCI128164 PMC6763238 31483289

[69] Zhou L Miao K Yin B Li H Fan J Zhu Y. Cardioprotective role of myeloid-derived suppressor cells in heart failure. Circulation (2018) 138(2):181–97. doi: 10.1161/CIRCULATIONAHA.117.030811 29437117

[70] Scheurer J Reisser T Leithauser F Messmann JJ Holzmann K Debatin KM. Rapamycin-based graft-Versus-Host disease prophylaxis increases the immunosuppressivity of myeloid-derived suppressor cells without affecting T cells and anti-tumor cytotoxicity. Clin Exp Immunol (2020) 202(3):407–22. doi: 10.1111/cei.13496 PMC7670162 32681646

[71] Wei C Wang Y Ma L Wang X Chi H Zhang S. Rapamycin nano-micelle ophthalmic solution reduces corneal allograft rejection by potentiating myeloid-derived suppressor cells' function. Front Immunol (2018) 9:2283. doi: 10.3389/fimmu.2018.02283 30349533 PMC6186809

[72] Zhang Y Bi Y Yang H Chen X Liu H Lu Y. Mtor limits the recruitment of Cd11b+Gr1+Ly6chigh myeloid-derived suppressor cells in protecting against murine immunological hepatic injury. J Leukoc Biol (2014) 95(6):961–70. doi: 10.1189/jlb.0913473 24569105

[73] Deissler A Della Penna A van Geffen C Gonzalez-Menendez I Quintanilla-Martinez L Gunther A. Rapamycin delays allograft rejection in obese graft recipients through induction of myeloid-derived suppressor cells. Immunol Lett (2021) 236:1–11. doi: 10.1016/j.imlet.2021.05.003 34015361

[74] Li Y Xu Y Liu X Yan X Lin Y Tan Q. Mtor inhibitor Ink128 promotes wound healing by regulating mdscs. Stem Cell Res Ther (2021) 12(1):170. doi: 10.1186/s13287-021-02206-y 33691762 PMC7944919

[75] Li J Chen J Zhang M Zhang C Wu R Yang T. The mtor deficiency in monocytic myeloid-derived suppressor cells protects mouse cardiac allografts by inducing allograft tolerance. Front Immunol (2021) 12:661338. doi: 10.3389/fimmu.2021.661338 33897705 PMC8062712

[76] Li X Li Y Yu Q Xu L Fu S Wei C. Mtor signaling regulates the development and therapeutic efficacy of pmn-mdscs in acute gvhd. Front Cell Dev Biol (2021) 9:741911. doi: 10.3389/fcell.2021.741911 35004668 PMC8733691

[77] Jia A Wang Y Wang Y Li Y Yang Q Cao Y. The kinase Akt1 potentiates the suppressive functions of myeloid-derived suppressor cells in inflammation and cancer. Cell Mol Immunol (2021) 18(4):1074–76. doi: 10.1038/s41423-020-00610-7 PMC8115558 33462382

[78] Li YM Liu ZY Wang JC Yu JM Li ZC Yang HJ. Receptor-interacting protein kinase 3 deficiency recruits myeloid-derived suppressor cells to hepatocellular carcinoma through the chemokine (C-X-C motif) ligand 1-chemokine (C-X-C motif) receptor 2 axis. Hepatology (2019) 70(5):1564–81. doi: 10.1002/hep.30676 PMC6900048 31021443

[79] Liu M Zhang H Zhang L Liu X Zhou S Wang X. Rip3 blockade prevents immune-mediated hepatitis through a myeloid-derived suppressor cell dependent mechanism. Int J Biol Sci (2022) 18(1):199–213. doi: 10.7150/ijbs.65402 34975327 PMC8692153

[80] Lusthaus M Mazkereth N Donin N Fishelson Z. Receptor-interacting protein kinases 1 and 3, and mixed lineage kinase domain-like protein are activated by sublytic complement and participate in complement-dependent cytotoxicity. Front Immunol (2018) 9:306. doi: 10.3389/fimmu.2018.00306 29527209 PMC5829068

[81] Sendo S Saegusa J Yamada H Nishimura K Morinobu A. Tofacitinib facilitates the expansion of myeloid-derived suppressor cells and ameliorates interstitial lung disease in skg mice. Arthritis Res Ther (2019) 21(1):184. doi: 10.1186/s13075-019-1963-2 31387650 PMC6685227

[82] Nishimura K Saegusa J Matsuki F Akashi K Kageyama G Morinobu A. Tofacitinib facilitates the expansion of myeloid-derived suppressor cells and ameliorates arthritis in skg mice. Arthritis Rheumatol (2015) 67(4):893–902. doi: 10.1002/art.39007 25545152

[83] Li J Yang F Wei F Ren X. The role of toll-like receptor 4 in tumor microenvironment. Oncotarget (2017) 8(39):66656–67. doi: 10.18632/oncotarget.19105 PMC5630445 29029545

[84] Locker KCS Kachapati K Wu Y Bednar KJ Adams D Patel C. Endosomal sequestration of Tlr4 antibody induces myeloid-derived suppressor cells and reverses acute type 1 diabetes. Diabetes (2022) 71(3):470–82. doi: 10.2337/db21-0426 PMC8893939 35040474

[85] Zhan X Jiang X He Q Zhong L Wang Y Huang Y. Pam2 lipopeptides enhance the immunosuppressive activity of monocytic myeloid-derived suppressor cells by Stat3 signal in chronic inflammation. Cent Eur J Immunol (2022) 47(1):30–40. doi: 10.5114/ceji.2022.113086 35600157 PMC9115589

[86] Ribechini E Eckert I Beilhack A Du Plessis N Walzl G Schleicher U. Heat-killed mycobacterium tuberculosis prime-boost vaccination induces myeloid-derived suppressor cells with spleen dendritic cell-killing capability. JCI Insight (2019) 5(13):e128664. doi: 10.1172/jci.insight.128664 PMC6629241 31162143

[87] Alpdundar Bulut E Bayyurt Kocabas B Yazar V Aykut G Guler U Salih B. Human gut commensal membrane vesicles modulate inflammation by generating M2-like macrophages and myeloid-derived suppressor cells. J Immunol (2020) 205(10):2707–18. doi: 10.4049/jimmunol.2000731 33028617

[88] Elliott DM Singh N Nagarkatti M Nagarkatti PS. Cannabidiol attenuates experimental autoimmune encephalomyelitis model of multiple sclerosis through induction of myeloid-derived suppressor cells. Front Immunol (2018) 9:1782. doi: 10.3389/fimmu.2018.01782 30123217 PMC6085417

[89] Hegde VL Nagarkatti PS Nagarkatti M. Role of myeloid-derived suppressor cells in amelioration of experimental autoimmune hepatitis following activation of Trpv1 receptors by cannabidiol. PloS One (2011) 6(4):e18281. doi: 10.1371/journal.pone.0018281 21483776 PMC3069975

[90] Namkoong H Ishii M Fujii H Yagi K Asami T Asakura T. Clarithromycin expands Cd11b+Gr-1+ cells *Via* the Stat3/Bv8 axis to ameliorate lethal endotoxic shock and post-influenza bacterial pneumonia. PloS Pathog (2018) 14(4):e1006955. doi: 10.1371/journal.ppat.1006955 29621339 PMC5886688

[91] Chang S Kim YH Kim YJ Kim YW Moon S Lee YY. Taurodeoxycholate increases the number of myeloid-derived suppressor cells that ameliorate sepsis in mice. Front Immunol (2018) 9:1984. doi: 10.3389/fimmu.2018.01984 30279688 PMC6153344

[92] Bashor CJ Hilton IB Bandukwala H Smith DM Veiseh O. Engineering the next generation of cell-based therapeutics. Nat Rev Drug Discovery (2022) (online ahead of print):1–21. doi: 10.1038/s41573-022-00476-6 PMC9149674 35637318

[93] Lee YS Zhang T Saxena V Li L Piao W Bromberg JS. Myeloid-derived suppressor cells expand after transplantation and their augmentation increases graft survival. Am J Transplant (2020) 20(9):2343–55. doi: 10.1111/ajt.15879 32282980

[94] Ren Y Dong X Zhao H Feng J Chen B Zhou Y. Myeloid-derived suppressor cells improve corneal graft survival through suppressing angiogenesis and lymphangiogenesis. Am J Transplant (2021) 21(2):552–66. doi: 10.1111/ajt.16291 32892499

[95] Mildner A Mack M Schmidt H Bruck W Djukic M Zabel MD. Ccr2+Ly-6chi monocytes are crucial for the effector phase of autoimmunity in the central nervous system. Brain (2009) 132(Pt 9):2487–500. doi: 10.1093/brain/awp144 19531531

[96] Jeong HJ Lee HJ Ko JH Cho BJ Park SY Park JW. Myeloid-derived suppressor cells mediate inflammation resolution in humans and mice with autoimmune uveoretinitis. J Immunol (2018) 200(4):1306–15. doi: 10.4049/jimmunol.1700617 29311360

[97] Park MJ Lee SH Kim EK Lee EJ Park SH Kwok SK. Myeloid-derived suppressor cells induce the expansion of regulatory b cells and ameliorate autoimmunity in the sanroque mouse model of systemic lupus erythematosus. Arthritis Rheumatol (2016) 68(11):2717–27. doi: 10.1002/art.39767 27214349

[98] Li Y Tu Z Qian S Fung JJ Markowitz SD Kusner LL. Myeloid-derived suppressor cells as a potential therapy for experimental autoimmune myasthenia gravis. J Immunol (2014) 193(5):2127–34. doi: 10.4049/jimmunol.1400857 PMC4784709 25057008

[99] Chiasson VL Bounds KR Chatterjee P Manandhar L Pakanati AR Hernandez M. Myeloid-derived suppressor cells ameliorate cyclosporine a-induced hypertension in mice. Hypertension (2018) 71(1):199–207. doi: 10.1161/HYPERTENSIONAHA.117.10306 29133357 PMC5730469

[100] van Geffen C Deissler A Beer-Hammer S Nurnberg B Handgretinger R Renz H. Myeloid-derived suppressor cells dampen airway inflammation through prostaglandin E2 receptor 4. Front Immunol (2021) 12:695933. doi: 10.3389/fimmu.2021.695933 34322123 PMC8311661

[101] Park MY Lim BG Kim SY Sohn HJ Kim S Kim TG. Gm-csf promotes the expansion and differentiation of cord blood myeloid-derived suppressor cells, which attenuate xenogeneic graft-Vs.-Host disease. Front Immunol (2019) 10:183. doi: 10.3389/fimmu.2019.00183 30863394 PMC6399310

[102] Hsieh CC Lin CL He JT Chiang M Wang Y Tsai YC. Administration of cytokine-induced myeloid-derived suppressor cells ameliorates renal fibrosis in diabetic mice. Stem Cell Res Ther (2018) 9(1):183. doi: 10.1186/s13287-018-0915-0 29973247 PMC6032782

[103] Yang F Li Y Zou W Xu Y Wang H Wang W. Adoptive transfer of ifn-Gamma-Induced m-mdscs promotes immune tolerance to allografts through inos pathway. Inflamm Res (2019) 68(7):545–55. doi: 10.1007/s00011-019-01237-9 31055608

[104] Yang F Li Y Wu T Na N Zhao Y Li W. Tnfalpha-induced m-mdscs promote transplant immune tolerance *Via* nitric oxide. J Mol Med (Berl) (2016) 94(8):911–20. doi: 10.1007/s00109-016-1398-z 26936474

[105] Kurko J Vida A Ocsko T Tryniszewska B Rauch TA Glant TT. Suppression of proteoglycan-induced autoimmune arthritis by myeloid-derived suppressor cells generated in vitro from murine bone marrow. PloS One (2014) 9(11):e111815. doi: 10.1371/journal.pone.0111815 25369029 PMC4219784

[106] Wang H Ji J Zhuang Y Zhou X Zhao Y Zhang X. Pma induces the differentiation of monocytes into immunosuppressive mdscs. Clin Exp Immunol (2021) 206(2):216–25. doi: 10.1111/cei.13657 PMC8506123 34453319

[107] Hsu CY Lin YC Chang LY Huang SK Huang CH Yang CK. Therapeutic role of inducible nitric oxide synthase expressing myeloid-derived suppressor cells in acetaminophen-induced murine liver failure. Front Immunol (2020) 11:574839. doi: 10.3389/fimmu.2020.574839 33250891 PMC7673381

[108] Czock D Keller F Rasche FM Haussler U. Pharmacokinetics and Pharmacodynamics of Systemically Administered Glucocorticoids. Clin Pharmacokinet (2005) 44(1):61–98. doi: 10.2165/00003088-200544010-00003. 15634032

[109] Wang Y Ding Y Guo N Wang S. Mdscs: Key criminals of tumor pre-metastatic niche formation. Front Immunol (2019) 10:172. doi: 10.3389/fimmu.2019.00172 30792719 PMC6374299

[110] Yuan K Zhu Q Lu Q Jiang H Zhu M Li X. Quercetin alleviates rheumatoid arthritis by inhibiting neutrophil inflammatory activities. J Nutr Biochem (2020) 84:108454. doi: 10.1016/j.jnutbio.2020.108454 32679549

[111] Javadi F Ahmadzadeh A Eghtesadi S Aryaeian N Zabihiyeganeh M Rahimi Foroushani A. The effect of quercetin on inflammatory factors and clinical symptoms in women with rheumatoid arthritis: A double-blind, randomized controlled trial. J Am Coll Nutr (2017) 36(1):9–15. doi: 10.1080/07315724.2016.1140093 27710596

[112] Comalada M Camuesco D Sierra S Ballester I Xaus J Galvez J. *In vivo* quercitrin anti-inflammatory effect involves release of quercetin, which inhibits inflammation through down-regulation of the nf-kappab pathway. Eur J Immunol (2005) 35(2):584–92. doi: 10.1002/eji.200425778 15668926

[113] Lalive PH Neuhaus O Benkhoucha M Burger D Hohlfeld R Zamvil SS. Glatiramer acetate in the treatment of multiple sclerosis: Emerging concepts regarding its mechanism of action. CNS Drugs (2011) 25(5):401–14. doi: 10.2165/11588120-000000000-00000 PMC3963480 21476611

[114] Shao B Wei X Luo M Yu J Tong A Ma X. Inhibition of A20 expression in tumor microenvironment exerts anti-tumor effect through inducing myeloid-derived suppressor cells apoptosis. Sci Rep (2015) 5:16437. doi: 10.1038/srep16437 26561336 PMC4642332

[115] Trillo-Tinoco J Sierra RA Mohamed E Cao Y de Mingo-Pulido A Gilvary DL. Ampk alpha-1 intrinsically regulates the function and differentiation of tumor myeloid-derived suppressor cells. Cancer Res (2019) 79(19):5034–47. doi: 10.1158/0008-5472.CAN-19-0880 PMC6774829 31409640

[116] Qin G Lian J Huang L Zhao Q Liu S Zhang Z. Metformin blocks myeloid-derived suppressor cell accumulation through ampk-Dach1-Cxcl1 axis. Oncoimmunology (2018) 7(7):e1442167. doi: 10.1080/2162402X.2018.1442167 29900050 PMC5993496

[117] Li L Wang L Li J Fan Z Yang L Zhang Z. Metformin-induced reduction of Cd39 and Cd73 blocks myeloid-derived suppressor cell activity in patients with ovarian cancer. Cancer Res (2018) 78(7):1779–91. doi: 10.1158/0008-5472.CAN-17-2460 PMC5882589 29374065

[118] Xu P Yin K Tang X Tian J Zhang Y Ma J. Metformin inhibits the function of granulocytic myeloid-derived suppressor cells in tumor-bearing mice. BioMed Pharmacother (2019) 120:109458. doi: 10.1016/j.biopha.2019.109458 31550676

[119] Bak SP Alonso A Turk MJ Berwin B. Murine ovarian cancer vascular leukocytes require arginase-1 activity for T cell suppression. Mol Immunol (2008) 46(2):258–68. doi: 10.1016/j.molimm.2008.08.266 PMC2613193 18824264

[120] Ye C Geng Z Dominguez D Chen S Fan J Qin L. Targeting ornithine decarboxylase by alpha-difluoromethylornithine inhibits tumor growth by impairing myeloid-derived suppressor cells. J Immunol (2016) 196(2):915–23. doi: 10.4049/jimmunol.1500729 PMC4707077 26663722

[121] Steggerda SM Bennett MK Chen J Emberley E Huang T Janes JR. Inhibition of arginase by cb-1158 blocks myeloid cell-mediated immune suppression in the tumor microenvironment. J Immunother Cancer (2017) 5(1):101. doi: 10.1186/s40425-017-0308-4 29254508 PMC5735564

[122] Cao Y Feng Y Zhang Y Zhu X Jin F. L-arginine supplementation inhibits the growth of breast cancer by enhancing innate and adaptive immune responses mediated by suppression of mdscs in vivo. BMC Cancer (2016) 16:343. doi: 10.1186/s12885-016-2376-0 27246354 PMC4888479

[123] Satoh Y Kotani H Iida Y Taniura T Notsu Y Harada M. Supplementation of l-arginine boosts the therapeutic efficacy of anticancer chemoimmunotherapy. Cancer Sci (2020) 111(7):2248–58. doi: 10.1111/cas.14490 PMC7484823 32426941

[124] Yin T Zhao ZB Guo J Wang T Yang JB Wang C. Aurora a inhibition eliminates myeloid cell-mediated immunosuppression and enhances the efficacy of anti-Pd-L1 therapy in breast cancer. Cancer Res (2019) 79(13):3431–44. doi: 10.1158/0008-5472.CAN-18-3397 30902796

[125] Hu X Bardhan K Paschall AV Yang D Waller JL Park MA. Deregulation of apoptotic factors bcl-xl and bax confers apoptotic resistance to myeloid-derived suppressor cells and contributes to their persistence in cancer. J Biol Chem (2013) 288(26):19103–15. doi: 10.1074/jbc.M112.434530 PMC3696683 23677993

[126] Fjaestad KY Romer AMA Goitea V Johansen AZ Thorseth ML Carretta M. Blockade of beta-adrenergic receptors reduces cancer growth and enhances the response to anti-Ctla4 therapy by modulating the tumor microenvironment. Oncogene (2022) 41(9):1364–75. doi: 10.1038/s41388-021-02170-0 PMC8881216 35017664

[127] Mohammadpour H MacDonald CR McCarthy PL Abrams SI Repasky EA. Beta2-adrenergic receptor signaling regulates metabolic pathways critical to myeloid-derived suppressor cell function within the tme. Cell Rep (2021) 37(4):109883. doi: 10.1016/j.celrep.2021.109883 34706232 PMC8601406

[128] Calvani M Bruno G Dal Monte M Nassini R Fontani F Casini A. Beta3 -adrenoceptor as a potential immuno-suppressor agent in melanoma. Br J Pharmacol (2019) 176(14):2509–24. doi: 10.1111/bph.14660 PMC6592854 30874296

[129] Radwan FF Hossain A God JM Leaphart N Elvington M Nagarkatti M. Reduction of myeloid-derived suppressor cells and lymphoma growth by a natural triterpenoid. J Cell Biochem (2015) 116(1):102–14. doi: 10.1002/jcb.24946 PMC5012903 25142864

[130] Terlizzi M Di Crescenzo VG Perillo G Galderisi A Pinto A Sorrentino R. Pharmacological inhibition of caspase-8 limits lung tumour outgrowth. Br J Pharmacol (2015) 172(15):3917–28. doi: 10.1111/bph.13176 PMC4523345 25917370

[131] Fan Q Gu D Liu H Yang L Zhang X Yoder MC. Defective tgf-beta signaling in bone marrow-derived cells prevents hedgehog-induced skin tumors. Cancer Res (2014) 74(2):471–83. doi: 10.1158/0008-5472.CAN-13-2134-T PMC3963525 24282281

[132] Mu XY Wang RJ Yao ZX Zheng Z Jiang JT Tan MY. Rs 504393 inhibits m-mdscs recruiting in immune microenvironment of bladder cancer after gemcitabine treatment. Mol Immunol (2019) 109:140–8. doi: 10.1016/j.molimm.2019.02.014 30951933

[133] Wang Y Zhang X Yang L Xue J Hu G. Blockade of Ccl2 enhances immunotherapeutic effect of anti-Pd1 in lung cancer. J Bone Oncol (2018) 11:27–32. doi: 10.1016/j.jbo.2018.01.002 29892522 PMC5993943

[134] ?>Masuda T Noda M Kogawa T Kitagawa D Hayashi N Jomori T. Phase I dose-escalation trial to repurpose propagermanium, an oral Ccl2 inhibitor, in patients with breast cancer. Cancer Sci (2020) 111(3):924–31. doi: 10.1111/cas.14306 PMC7060487 31943636

[135] Schlecker E Stojanovic A Eisen C Quack C Falk CS Umansky V. Tumor-infiltrating monocytic myeloid-derived suppressor cells mediate Ccr5-dependent recruitment of regulatory T cells favoring tumor growth. J Immunol (2012) 189(12):5602–11. doi: 10.4049/jimmunol.1201018 23152559

[136] Zhang Y Lv D Kim HJ Kurt RA Bu W Li Y. A novel role of hematopoietic Ccl5 in promoting triple-negative mammary tumor progression by regulating generation of myeloid-derived suppressor cells. Cell Res (2013) 23(3):394–408. doi: 10.1038/cr.2012.178 23266888 PMC3587709

[137] Tang Q Jiang J Liu J. Ccr5 blockade suppresses melanoma development through inhibition of il-6-Stat3 pathway *Via* upregulation of Socs3. Inflammation (2015) 38(6):2049–56. doi: 10.1007/s10753-015-0186-1 26047948

[138] Ban Y Mai J Li X Mitchell-Flack M Zhang T Zhang L. Targeting autocrine Ccl5-Ccr5 axis reprograms immunosuppressive myeloid cells and reinvigorates antitumor immunity. Cancer Res (2017) 77(11):2857–68. doi: 10.1158/0008-5472.CAN-16-2913 PMC5484057 28416485

[139] Zhou J Liu M Sun H Feng Y Xu L Chan AWH. Hepatoma-intrinsic ccrk inhibition diminishes myeloid-derived suppressor cell immunosuppression and enhances immune-checkpoint blockade efficacy. Gut (2018) 67(5):931–44. doi: 10.1136/gutjnl-2017-314032 PMC5961939 28939663

[140] Eksioglu EA Chen X Heider KH Rueter B McGraw KL Basiorka AA. Novel therapeutic approach to improve hematopoiesis in low risk mds by targeting mdscs with the fc-engineered Cd33 antibody bi 836858. Leukemia (2017) 31(10):2172–80. doi: 10.1038/leu.2017.21 PMC5552472 28096534

[141] Jitschin R Saul D Braun M Tohumeken S Volkl S Kischel R. Cd33/Cd3-bispecific T-cell engaging (Bite(R)) antibody construct targets monocytic aml myeloid-derived suppressor cells. J Immunother Cancer (2018) 6(1):116. doi: 10.1186/s40425-018-0432-9 30396365 PMC6217777

[142] Cheng P Chen X Dalton R Calescibetta A So T Gilvary D. Immunodepletion of mdsc by Amv564, a novel bivalent, bispecific Cd33/Cd3 T cell engager, ex vivo in mds and melanoma. Mol Ther (2022) 30(6):2315–26. doi: 10.1016/j.ymthe.2022.02.005 PMC9171150 35150889

[143] Pan PY Ma G Weber KJ Ozao-Choy J Wang G Yin B. Immune stimulatory receptor Cd40 is required for T-cell suppression and T regulatory cell activation mediated by myeloid-derived suppressor cells in cancer. Cancer Res (2010) 70(1):99–108. doi: 10.1158/0008-5472.CAN-09-1882 19996287 PMC2805053

[144] Shen XZ Okwan-Duodu D Blackwell WL Ong FS Janjulia T Bernstein EA. Myeloid expression of angiotensin-converting enzyme facilitates myeloid maturation and inhibits the development of myeloid-derived suppressor cells. Lab Invest (2014) 94(5):536–44. doi: 10.1038/labinvest.2014.41 PMC4221240 24614194

[145] Weiss JM Subleski JJ Back T Chen X Watkins SK Yagita H. Regulatory T cells and myeloid-derived suppressor cells in the tumor microenvironment undergo fas-dependent cell death during il-2/Alphacd40 therapy. J Immunol (2014) 192(12):5821–9. doi: 10.4049/jimmunol.1400404 PMC4048774 24808361

[146] Buzzelli JN Pavlic DI Chalinor HV O'Connor L Menheniott TR Giraud AS. Il-1rt1 signaling antagonizes il-11 induced Stat3 dependent cardiac and antral stomach tumor development through myeloid cell enrichment. Oncotarget (2015) 6(2):679–95. doi: 10.18632/oncotarget.2707 PMC4359248 25528766

[147] Li T Li X Zamani A Wang W Lee CN Li M. C-rel is a myeloid checkpoint for cancer immunotherapy. Nat Cancer (2020) 1(5):507–17. doi: 10.1038/s43018-020-0061-3 PMC7808269 33458695

[148] Garcia AJ Ruscetti M Arenzana TL Tran LM Bianci-Frias D Sybert E. Pten null prostate epithelium promotes localized myeloid-derived suppressor cell expansion and immune suppression during tumor initiation and progression. Mol Cell Biol (2014) 34(11):2017–28. doi: 10.1128/MCB.00090-14 PMC4019050 24662052

[149] Mok S Tsoi J Koya RC Hu-Lieskovan S West BL Bollag G. Inhibition of colony stimulating factor-1 receptor improves antitumor efficacy of braf inhibition. BMC Cancer (2015) 15:356. doi: 10.1186/s12885-015-1377-8 25939769 PMC4432503

[150] Mao Y Eissler N Blanc KL Johnsen JI Kogner P Kiessling R. Targeting suppressive myeloid cells potentiates checkpoint inhibitors to control spontaneous neuroblastoma. Clin Cancer Res (2016) 22(15):3849–59. doi: 10.1158/1078-0432.CCR-15-1912 26957560

[151] Holmgaard RB Zamarin D Lesokhin A Merghoub T Wolchok JD. Targeting myeloid-derived suppressor cells with colony stimulating factor-1 receptor blockade can reverse immune resistance to immunotherapy in indoleamine 2,3-Dioxygenase-Expressing tumors. EBioMedicine (2016) 6:50–8. doi: 10.1016/j.ebiom.2016.02.024 PMC4856741 27211548

[152] Yu F Shi Y Wang J Li J Fan D Ai W. Deficiency of kruppel-like factor Klf4 in mammary tumor cells inhibits tumor growth and pulmonary metastasis and is accompanied by compromised recruitment of myeloid-derived suppressor cells. Int J Cancer (2013) 133(12):2872–83. doi: 10.1002/ijc.28302 PMC3796127 23737434

[153] Highfill SL Cui Y Giles AJ Smith JP Zhang H Morse E. Disruption of Cxcr2-mediated mdsc tumor trafficking enhances anti-Pd1 efficacy. Sci Transl Med (2014) 6(237):237ra67. doi: 10.1126/scitranslmed.3007974 PMC6980372 24848257

[154] Wang G Lu X Dey P Deng P Wu CC Jiang S. Targeting yap-dependent mdsc infiltration impairs tumor progression. Cancer Discovery (2016) 6(1):80–95. doi: 10.1158/2159-8290.CD-15-0224 26701088 PMC4707102

[155] Dong Y Fu R Chen J Zhang K Ji M Wang M. Discovery of benzocyclic sulfone derivatives as potent Cxcr2 antagonists for cancer immunotherapy. J Med Chem (2021) 64(22):16626–40. doi: 10.1021/acs.jmedchem.1c01219 34676759

[156] Wang D Sun H Wei J Cen B DuBois RN. Cxcl1 is critical for premetastatic niche formation and metastasis in colorectal cancer. Cancer Res (2017) 77(13):3655–65. doi: 10.1158/0008-5472.CAN-16-3199 PMC5877403 28455419

[157] Shi H Han X Sun Y Shang C Wei M Ba X. Chemokine (C-X-C motif) ligand 1 and Cxcl2 produced by tumor promote the generation of monocytic myeloid-derived suppressor cells. Cancer Sci (2018) 109(12):3826–39. doi: 10.1111/cas.13809 PMC6272093 30259595

[158] Greene S Robbins Y Mydlarz WK Huynh AP Schmitt NC Friedman J. Inhibition of mdsc trafficking with sx-682, a Cxcr1/2 inhibitor, enhances nk-cell immunotherapy in head and neck cancer models. Clin Cancer Res (2020) 26(6):1420–31. doi: 10.1158/1078-0432.CCR-19-2625 PMC7073293 31848188

[159] Dubeykovskaya Z Si Y Chen X Worthley DL Renz BW Urbanska AM. Neural innervation stimulates splenic Tff2 to arrest myeloid cell expansion and cancer. Nat Commun (2016) 7:10517. doi: 10.1038/ncomms10517 26841680 PMC4742920

[160] Hwang HS Han AR Lee JY Park GS Min WS Kim HJ. Enhanced anti-leukemic effects through induction of immunomodulating microenvironment by blocking Cxcr4 and pd-L1 in an aml mouse model. Immunol Invest (2019) 48(1):96–105. doi: 10.1080/08820139.2018.1497057 30204524

[161] Sun R Luo H Su J Di S Zhou M Shi B. Olaparib suppresses mdsc recruitment *Via* Sdf1alpha/Cxcr4 axis to improve the anti-tumor efficacy of car-T cells on breast cancer in mice. Mol Ther (2021) 29(1):60–74. doi: 10.1016/j.ymthe.2020.09.034 33010818 PMC7791086

[162] Bockorny B Semenisty V Macarulla T Borazanci E Wolpin BM Stemmer SM. Bl-8040, a Cxcr4 antagonist, in combination with pembrolizumab and chemotherapy for pancreatic cancer: The combat trial. Nat Med (2020) 26(6):878–85. doi: 10.1038/s41591-020-0880-x 32451495

[163] Ghonim MA Ibba SV Tarhuni AF Errami Y Luu HH Dean MJ. Targeting parp-1 with metronomic therapy modulates mdsc suppressive function and enhances anti-Pd-1 immunotherapy in colon cancer. J Immunother Cancer (2021) 9(1):e001643. doi: 10.1136/jitc-2020-001643 33495297 PMC7839867

[164] Travelli C Consonni FM Sangaletti S Storto M Morlacchi S Grolla AA. Nicotinamide phosphoribosyltransferase acts as a metabolic gate for mobilization of myeloid-derived suppressor cells. Cancer Res (2019) 79(8):1938–51. doi: 10.1158/0008-5472.CAN-18-1544 30777853

[165] Wu J Zhang R Tang N Gong Z Zhou J Chen Y. Dopamine inhibits the function of gr-1+Cd115+ myeloid-derived suppressor cells through D1-like receptors and enhances anti-tumor immunity. J Leukoc Biol (2015) 97(1):191–200. doi: 10.1189/jlb.5A1113-626RR 25341727 PMC4377827

[166] Hoeppner LH Wang Y Sharma A Javeed N Van Keulen VP Wang E. Dopamine D2 receptor agonists inhibit lung cancer progression by reducing angiogenesis and tumor infiltrating myeloid derived suppressor cells. Mol Oncol (2015) 9(1):270–81. doi: 10.1016/j.molonc.2014.08.008 PMC4277897 25226814

[167] Albeituni SH Ding C Liu M Hu X Luo F Kloecker G. Yeast-derived particulate beta-glucan treatment subverts the suppression of myeloid-derived suppressor cells (Mdsc) by inducing polymorphonuclear mdsc apoptosis and monocytic mdsc differentiation to apc in cancer. J Immunol (2016) 196(5):2167–80. doi: 10.4049/jimmunol.1501853 PMC4761495 26810222

[168] D'Amico L Mahajan S Capietto AH Yang Z Zamani A Ricci B. Dickkopf-related protein 1 (Dkk1) regulates the accumulation and function of myeloid derived suppressor cells in cancer. J Exp Med (2016) 213(5):827–40. doi: 10.1084/jem.20150950 PMC4854727 27045006

[169] Park MS Yang AY Lee JE Kim SK Roe JS Park MS. Galnt3 suppresses lung cancer by inhibiting myeloid-derived suppressor cell infiltration and angiogenesis in a tnfr and c-met pathway-dependent manner. Cancer Lett (2021) 521:294–307. doi: 10.1016/j.canlet.2021.08.015 34416337

[170] Sinha P Clements VK Bunt SK Albelda SM Ostrand-Rosenberg S. Cross-talk between myeloid-derived suppressor cells and macrophages subverts tumor immunity toward a type 2 response. J Immunol (2007) 179(2):977–83. doi: 10.4049/jimmunol.179.2.977 17617589

[171] Ghansah T Vohra N Kinney K Weber A Kodumudi K Springett G. Dendritic cell immunotherapy combined with gemcitabine chemotherapy enhances survival in a murine model of pancreatic carcinoma. Cancer Immunol Immunother (2013) 62(6):1083–91. doi: 10.1007/s00262-013-1407-9 PMC3666559 23604104

[172] Sasso MS Lollo G Pitorre M Solito S Pinton L Valpione S. Low dose gemcitabine-loaded lipid nanocapsules target monocytic myeloid-derived suppressor cells and potentiate cancer immunotherapy. Biomaterials (2016) 96:47–62. doi: 10.1016/j.biomaterials.2016.04.010 27135716

[173] Peereboom DM Alban TJ Grabowski MM Alvarado AG Otvos B Bayik D. Metronomic capecitabine as an immune modulator in glioblastoma patients reduces myeloid-derived suppressor cells. JCI Insight (2019) 4(22):e130748. doi: 10.1172/jci.insight.130748 PMC6948860 31600167

[174] Yuan SJ Xu YH Wang C An HC Xu HZ Li K. Doxorubicin-Polyglycerol-Nanodiamond conjugate is a cytostatic agent that evades chemoresistance and reverses cancer-induced immunosuppression in triple-negative breast cancer. J Nanobiotechnol (2019) 17(1):110. doi: 10.1186/s12951-019-0541-8 PMC6798483 31623629

[175] Vincent J Mignot G Chalmin F Ladoire S Bruchard M Chevriaux A. 5-fluorouracil selectively kills tumor-associated myeloid-derived suppressor cells resulting in enhanced T cell-dependent antitumor immunity. Cancer Res (2010) 70(8):3052–61. doi: 10.1158/0008-5472.CAN-09-3690 20388795

[176] Kuroda H Mabuchi S Kozasa K Yokoi E Matsumoto Y Komura N. Pm01183 inhibits myeloid-derived suppressor cells in vitro and in vivo. Immunotherapy (2017) 9(10):805–17. doi: 10.2217/imt-2017-0046 28877631

[177] Huang X Cui S Shu Y. Cisplatin selectively downregulated the frequency and immunoinhibitory function of myeloid-derived suppressor cells in a murine B16 melanoma model. Immunol Res (2016) 64(1):160–70. doi: 10.1007/s12026-015-8734-1 26590944

[178] Jeanbart L Kourtis IC van der Vlies AJ Swartz MA Hubbell JA. 6-Thioguanine-Loaded polymeric micelles deplete myeloid-derived suppressor cells and enhance the efficacy of T cell immunotherapy in tumor-bearing mice. Cancer Immunol Immunother (2015) 64(8):1033–46. doi: 10.1007/s00262-015-1702-8 PMC4506469 25982370

[179] Terracina KP Graham LJ Payne KK Manjili MH Baek A Damle SR. DNA Methyltransferase inhibition increases efficacy of adoptive cellular immunotherapy of murine breast cancer. Cancer Immunol Immunother (2016) 65(9):1061–73. doi: 10.1007/s00262-016-1868-8 PMC4996686 27416831

[180] Chiu DK Tse AP Xu IM Di Cui J Lai RK Li LL. Hypoxia inducible factor hif-1 promotes myeloid-derived suppressor cells accumulation through Entpd2/Cd39l1 in hepatocellular carcinoma. Nat Commun (2017) 8(1):517. doi: 10.1038/s41467-017-00530-7 28894087 PMC5593860

[181] Albu DI Wang Z Huang KC Wu J Twine N Leacu S. Ep4 antagonism by E7046 diminishes myeloid immunosuppression and synergizes with treg-reducing il-2-Diphtheria toxin fusion protein in restoring anti-tumor immunity. Oncoimmunology (2017) 6(8):e1338239. doi: 10.1080/2162402x.2017.1338239 28920002 PMC5593700

[182] Peng S Hu P Xiao YT Lu W Guo D Hu S. Single-cell analysis reveals Ep4 as a target for restoring T-cell infiltration and sensitizing prostate cancer to immunotherapy. Clin Cancer Res (2022) 28(3):552–67. doi: 10.1158/1078-0432.CCR-21-0299 34740924

[183] Hossain F Al-Khami AA Wyczechowska D Hernandez C Zheng L Reiss K. Inhibition of fatty acid oxidation modulates immunosuppressive functions of myeloid-derived suppressor cells and enhances cancer therapies. Cancer Immunol Res (2015) 3(11):1236–47. doi: 10.1158/2326-6066.CIR-15-0036 PMC4636942 26025381

[184] Li J Srivastava RM Ettyreddy A Ferris RL. Cetuximab ameliorates suppressive phenotypes of myeloid antigen presenting cells in head and neck cancer patients. J Immunother Cancer (2015) 3:54. doi: 10.1186/s40425-015-0097-6 26579227 PMC4647471

[185] Yan J Kong LY Hu J Gabrusiewicz K Dibra D Xia X. Fgl2 as a multimodality regulator of tumor-mediated immune suppression and therapeutic target in gliomas. J Natl Cancer Inst (2015) 107(8):djv137. doi: 10.1093/jnci/djv137 25971300 PMC4554195

[186] Waight JD Hu Q Miller A Liu S Abrams SI. Tumor-derived G-csf facilitates neoplastic growth through a granulocytic myeloid-derived suppressor cell-dependent mechanism. PloS One (2011) 6(11):e27690. doi: 10.1371/journal.pone.0027690 22110722 PMC3218014

[187] Wei WC Lin SY Lan CW Huang YC Lin CY Hsiao PW. Inhibiting mdsc differentiation from bone marrow with phytochemical polyacetylenes drastically impairs tumor metastasis. Sci Rep (2016) 6:36663. doi: 10.1038/srep36663 27857157 PMC5114612

[188] Nefedova Y Fishman M Sherman S Wang X Beg AA Gabrilovich DI. Mechanism of all-trans retinoic acid effect on tumor-associated myeloid-derived suppressor cells. Cancer Res (2007) 67(22):11021–8. doi: 10.1158/0008-5472.Can-07-2593 18006848

[189] Chen X Eksioglu EA Zhou J Zhang L Djeu J Fortenbery N. Induction of myelodysplasia by myeloid-derived suppressor cells. J Clin Invest (2013) 123(11):4595–611. doi: 10.1172/JCI67580 PMC3809779 24216507

[190] Iclozan C Antonia S Chiappori A Chen DT Gabrilovich D. Therapeutic regulation of myeloid-derived suppressor cells and immune response to cancer vaccine in patients with extensive stage small cell lung cancer. Cancer Immunol Immunother (2013) 62(5):909–18. doi: 10.1007/s00262-013-1396-8 PMC3662237 23589106

[191] Long AH Highfill SL Cui Y Smith JP Walker AJ Ramakrishna S. Reduction of mdscs with all-trans retinoic acid improves car therapy efficacy for sarcomas. Cancer Immunol Res (2016) 4(10):869–80. doi: 10.1158/2326-6066.CIR-15-0230 PMC5050151 27549124

[192] Heine A Flores C Gevensleben H Diehl L Heikenwalder M Ringelhan M. Targeting myeloid derived suppressor cells with all-trans retinoic acid is highly time-dependent in therapeutic tumor vaccination. Oncoimmunology (2017) 6(8):e1338995. doi: 10.1080/2162402X.2017.1338995 28920004 PMC5593699

[193] Bauer R Udonta F Wroblewski M Ben-Batalla I Santos IM Taverna F. Blockade of myeloid-derived suppressor cell expansion with all-trans retinoic acid increases the efficacy of antiangiogenic therapy. Cancer Res (2018) 78(12):3220–32. doi: 10.1158/0008-5472.CAN-17-3415 29674477

[194] Tobin RP Jordan KR Robinson WA Davis D Borges VF Gonzalez R. Targeting myeloid-derived suppressor cells using all-trans retinoic acid in melanoma patients treated with ipilimumab. Int Immunopharmacol (2018) 63:282–91. doi: 10.1016/j.intimp.2018.08.007 PMC6134177 30121453

[195] Oh MH Sun IH Zhao L Leone RD Sun IM Xu W. Targeting glutamine metabolism enhances tumor-specific immunity by modulating suppressive myeloid cells. J Clin Invest (2020) 130(7):3865–84. doi: 10.1172/JCI131859 PMC7324212 32324593

[196] Morales JK Kmieciak M Knutson KL Bear HD Manjili MH. Gm-csf is one of the main breast tumor-derived soluble factors involved in the differentiation of Cd11b-Gr1- bone marrow progenitor cells into myeloid-derived suppressor cells. Breast Cancer Res Treat (2010) 123(1):39–49. doi: 10.1007/s10549-009-0622-8 PMC3095485 19898981

[197] Kapanadze T Gamrekelashvili J Ma C Chan C Zhao F Hewitt S. Regulation of accumulation and function of myeloid derived suppressor cells in different murine models of hepatocellular carcinoma. J Hepatol (2013) 59(5):1007–13. doi: 10.1016/j.jhep.2013.06.010 PMC3805787 23796475

[198] Takeuchi S Baghdadi M Tsuchikawa T Wada H Nakamura T Abe H. Chemotherapy-derived inflammatory responses accelerate the formation of immunosuppressive myeloid cells in the tissue microenvironment of human pancreatic cancer. Cancer Res (2015) 75(13):2629–40. doi: 10.1158/0008-5472.CAN-14-2921 25952647

[199] Wang HF Ning F Liu ZC Wu L Li ZQ Qi YF. Histone deacetylase inhibitors deplete myeloid-derived suppressor cells induced by 4t1 mammary tumors in vivo and in vitro. Cancer Immunol Immunother (2017) 66(3):355–66. doi: 10.1007/s00262-016-1935-1 PMC11028551 27915371

[200] Orillion A Hashimoto A Damayanti N Shen L Adelaiye-Ogala R Arisa S. Entinostat neutralizes myeloid-derived suppressor cells and enhances the antitumor effect of pd-1 inhibition in murine models of lung and renal cell carcinoma. Clin Cancer Res (2017) 23(17):5187–201. doi: 10.1158/1078-0432.CCR-17-0741 PMC5723438 28698201

[201] Xie Z Ikegami T Ago Y Okada N Tachibana M. Valproic acid attenuates Ccr2-dependent tumor infiltration of monocytic myeloid-derived suppressor cells, limiting tumor progression. Oncoimmunology (2020) 9(1):1734268. doi: 10.1080/2162402X.2020.1734268 32158627 PMC7051186

[202] Vila-Leahey A Oldford SA Marignani PA Wang J Haidl ID Marshall JS. Ranitidine modifies myeloid cell populations and inhibits breast tumor development and spread in mice. Oncoimmunology (2016) 5(7):e1151591. doi: 10.1080/2162402X.2016.1151591 27622015 PMC5006904

[203] Grauers Wiktorin H Nilsson MS Kiffin R Sander FE Lenox B Rydstrom A. Histamine targets myeloid-derived suppressor cells and improves the anti-tumor efficacy of pd-1/Pd-L1 checkpoint blockade. Cancer Immunol Immunother (2019) 68(2):163–74. doi: 10.1007/s00262-018-2253-6 PMC6394491 30315349

[204] Parker KH Sinha P Horn LA Clements VK Yang H Li J. Hmgb1 enhances immune suppression by facilitating the differentiation and suppressive activity of myeloid-derived suppressor cells. Cancer Res (2014) 74(20):5723–33. doi: 10.1158/0008-5472.CAN-13-2347 PMC4199911 25164013

[205] Tian X Ma J Wang T Tian J Zhang Y Mao L. Long non-coding rna hoxa transcript antisense rna myeloid-specific 1-Hoxa1 axis downregulates the immunosuppressive activity of myeloid-derived suppressor cells in lung cancer. Front Immunol (2018) 9:473. doi: 10.3389/fimmu.2018.00473 29662483 PMC5890118

[206] Smith C Chang MY Parker KH Beury DW DuHadaway JB Flick HE. Ido is a nodal pathogenic driver of lung cancer and metastasis development. Cancer Discov (2012) 2(8):722–35. doi: 10.1158/2159-8290.CD-12-0014 PMC3677576 22822050

[207] Holmgaard RB Zamarin D Li Y Gasmi B Munn DH Allison JP. Tumor-expressed ido recruits and activates mdscs in a treg-dependent manner. Cell Rep (2015) 13(2):412–24. doi: 10.1016/j.celrep.2015.08.077 PMC5013825 26411680

[208] Blair AB Kleponis J Thomas DL 2nd Muth ST Murphy AG Kim V. Ido1 inhibition potentiates vaccine-induced immunity against pancreatic adenocarcinoma. J Clin Invest (2019) 129(4):1742–55. doi: 10.1172/JCI124077 PMC6436883 30747725

[209] Schafer CC Wang Y Hough KP Sawant A Grant SC Thannickal VJ. Indoleamine 2,3-dioxygenase regulates anti-tumor immunity in lung cancer by metabolic reprogramming of immune cells in the tumor microenvironment. Oncotarget (2016) 7(46):75407–24. doi: 10.18632/oncotarget.12249 PMC5340181 27705910

[210] Li A Barsoumian HB Schoenhals JE Cushman TR Caetano MS Wang X. Indoleamine 2,3-dioxygenase 1 inhibition targets anti-Pd1-Resistant lung tumors by blocking myeloid-derived suppressor cells. Cancer Lett (2018) 431:54–63. doi: 10.1016/j.canlet.2018.05.005 29746927 PMC6027590

[211] Shi J Liu C Luo S Cao T Lin B Zhou M. Sting agonist and ido inhibitor combination therapy inhibits tumor progression in murine models of colorectal cancer. Cell Immunol (2021) 366:104384. doi: 10.1016/j.cellimm.2021.104384 34182334

[212] Nandre R Verma V Gaur P Patil V Yang X Ramlaoui Z. Ido-vaccine ablates immune-suppressive myeloid populations and enhances anti-tumor effects independent of tumor cell ido status. Cancer Immunol Res (2022) 10(5):571–80. doi: 10.1158/2326-6066.CIR-21-0457 PMC9381100 35290437

[213] Medina-Echeverz J Haile LA Zhao F Gamrekelashvili J Ma C Metais JY. Ifn-gamma regulates survival and function of tumor-induced Cd11b+ gr-1high myeloid derived suppressor cells by modulating the anti-apoptotic molecule Bcl2a1. Eur J Immunol (2014) 44(8):2457–67. doi: 10.1002/eji.201444497 PMC4140991 24810636

[214] Lu X Horner JW Paul E Shang X Troncoso P Deng P. Effective combinatorial immunotherapy for castration-resistant prostate cancer. Nature (2017) 543(7647):728–32. doi: 10.1038/nature21676 PMC5374023 28321130

[215] Tengesdal IW Menon DR Osborne DG Neff CP Powers NE Gamboni F. Targeting tumor-derived Nlrp3 reduces melanoma progression by limiting mdscs expansion. Proc Natl Acad Sci USA (2021) 118(10):e2000915118. doi: 10.1073/pnas.2000915118 33649199 PMC7958415

[216] Jiang J Wang Z Li Z Zhang J Wang C Xu X. Early exposure of high-dose interleukin-4 to tumor stroma reverses myeloid cell-mediated T-cell suppression. Gene Ther (2010) 17(8):991–9. doi: 10.1038/gt.2010.54 20410929

[217] Roth F de la Fuente AC Vella JL Zoso A Inverardi L Serafini P. Aptamer-mediated blockade of Il4ralpha triggers apoptosis of mdscs and limits tumor progression. Cancer Res (2012) 72(6):1373–83. doi: 10.1158/0008-5472.CAN-11-2772 22282665

[218] Chen MF Hsieh CC Chen WC Lai CH. Role of interleukin-6 in the radiation response of liver tumors. Int J Radiat Oncol Biol Phys (2012) 84(5):e621–30. doi: 10.1016/j.ijrobp.2012.07.2360 22975618

[219] Wu CT Hsieh CC Lin CC Chen WC Hong JH Chen MF. Significance of il-6 in the transition of hormone-resistant prostate cancer and the induction of myeloid-derived suppressor cells. J Mol Med (Berl) (2012) 90(11):1343–55. doi: 10.1007/s00109-012-0916-x 22660275

[220] Wu CT Chen MF Chen WC Hsieh CC. The role of il-6 in the radiation response of prostate cancer. Radiat Oncol (2013) 8:159. doi: 10.1186/1748-717X-8-159 23806095 PMC3717100

[221] Zhang T Guo W Yang Y Liu W Guo L Gu Y. Loss of shp-2 activity in Cd4+ T cells promotes melanoma progression and metastasis. Sci Rep (2013) 3:2845. doi: 10.1038/srep02845 24088816 PMC3789150

[222] Caetano MS Zhang H Cumpian AM Gong L Unver N Ostrin EJ. Il6 blockade reprograms the lung tumor microenvironment to limit the development and progression of K-Ras-Mutant lung cancer. Cancer Res (2016) 76(11):3189–99. doi: 10.1158/0008-5472.CAN-15-2840 PMC4891282 27197187

[223] Lee BR Kwon BE Hong EH Shim A Song JH Kim HM. Interleukin-10 attenuates tumour growth by inhibiting interleukin-6/Signal transducer and activator of transcription 3 signalling in myeloid-derived suppressor cells. Cancer Lett (2016) 381(1):156–64. doi: 10.1016/j.canlet.2016.07.012 27431309

[224] Dominguez C McCampbell KK David JM Palena C. Neutralization of il-8 decreases tumor pmn-mdscs and reduces mesenchymalization of claudin-low triple-negative breast cancer. JCI Insight (2017) 2(21):e94296. doi: 10.1172/jci.insight.94296 PMC5752275 29093275

[225] Bilusic M Heery CR Collins JM Donahue RN Palena C Madan RA. Phase I trial of humax-Il8 (Bms-986253), an anti-Il-8 monoclonal antibody, in patients with metastatic or unresectable solid tumors. J Immunother Cancer (2019) 7(1):240. doi: 10.1186/s40425-019-0706-x 31488216 PMC6729083

[226] Hart KM Byrne KT Molloy MJ Usherwood EM Berwin B. Il-10 immunomodulation of myeloid cells regulates a murine model of ovarian cancer. Front Immunol (2011) 2:29. doi: 10.3389/fimmu.2011.00029 22566819 PMC3342001

[227] Martinson HA Jindal S Durand-Rougely C Borges VF Schedin P. Wound healing-like immune program facilitates postpartum mammary gland involution and tumor progression. Int J Cancer (2015) 136(8):1803–13. doi: 10.1002/ijc.29181 PMC4324053 25187059

[228] Medina-Echeverz J Fioravanti J Zabala M Ardaiz N Prieto J Berraondo P. Successful colon cancer eradication after chemoimmunotherapy is associated with profound phenotypic change of intratumoral myeloid cells. J Immunol (2011) 186(2):807–15. doi: 10.4049/jimmunol.1001483 21148040

[229] Steding CE Wu ST Zhang Y Jeng MH Elzey BD Kao C. The role of interleukin-12 on modulating myeloid-derived suppressor cells, increasing overall survival and reducing metastasis. Immunology (2011) 133(2):221–38. doi: 10.1111/j.1365-2567.2011.03429.x PMC3088984 21453419

[230] Hall B Nakashima H Sun ZJ Sato Y Bian Y Husain SR. Targeting of interleukin-13 receptor Alpha2 for treatment of head and neck squamous cell carcinoma induced by conditional deletion of tgf-beta and pten signaling. J Transl Med (2013) 11:45. doi: 10.1186/1479-5876-11-45 23421960 PMC3598213

[231] Lim HX Hong HJ Cho D Kim TS. Il-18 enhances immunosuppressive responses by promoting differentiation into monocytic myeloid-derived suppressor cells. J Immunol (2014) 193(11):5453–60. doi: 10.4049/jimmunol.1401282 25362180

[232] Guan Y Zhang R Peng Z Dong D Wei G Wang Y. Inhibition of il-18-Mediated myeloid derived suppressor cell accumulation enhances anti-Pd1 efficacy against osteosarcoma cancer. J Bone Oncol (2017) 9:59–64. doi: 10.1016/j.jbo.2017.10.002 29226090 PMC5715437

[233] Nakamura K Kassem S Cleynen A Chretien ML Guillerey C Putz EM. Dysregulated il-18 is a key driver of immunosuppression and a possible therapeutic target in the multiple myeloma microenvironment. Cancer Cell (2018) 33(4):634–48 e5. doi: 10.1016/j.ccell.2018.02.007 29551594

[234] Lim HX Choi S Cho D Kim TS. Il-33 inhibits the differentiation and immunosuppressive activity of granulocytic myeloid-derived suppressor cells in tumor-bearing mice. Immunol Cell Biol (2017) 95(1):99–107. doi: 10.1038/icb.2016.72 27507556

[235] Capuano G Rigamonti N Grioni M Freschi M Bellone M. Modulators of arginine metabolism support cancer immunosurveillance. BMC Immunol (2009) 10:1. doi: 10.1186/1471-2172-10-1 19134173 PMC2648942

[236] Jayaraman P Parikh F Lopez-Rivera E Hailemichael Y Clark A Ma G. Tumor-expressed inducible nitric oxide synthase controls induction of functional myeloid-derived suppressor cells through modulation of vascular endothelial growth factor release. J Immunol (2012) 188(11):5365–76. doi: 10.4049/jimmunol.1103553 PMC3358566 22529296

[237] Nam S Kang K Cha JS Kim JW Lee HG Kim Y. Interferon regulatory factor 4 (Irf4) controls myeloid-derived suppressor cell (Mdsc) differentiation and function. J Leukoc Biol (2016) 100(6):1273–84. doi: 10.1189/jlb.1A0215-068RR 27601624

[238] Yang Q Xie H Li X Feng Y Xie S Qu J. Interferon regulatory factor 4 regulates the development of polymorphonuclear myeloid-derived suppressor cells through the transcription of c-myc in cancer. Front Immunol (2021) 12:627072. doi: 10.3389/fimmu.2021.627072 33708218 PMC7940347

[239] Parveen S Siddharth S Cheung LS Kumar A Shen J Murphy JR. Therapeutic targeting with dabil-4 depletes myeloid suppressor cells in 4t1 triple-negative breast cancer model. Mol Oncol (2021) 15(5):1330–44. doi: 10.1002/1878-0261.12938 PMC8096791 33682324

[240] Sierra RA Trillo-Tinoco J Mohamed E Yu L Achyut BR Arbab A. Anti-jagged immunotherapy inhibits mdscs and overcomes tumor-induced tolerance. Cancer Res (2017) 77(20):5628–38. doi: 10.1158/0008-5472.CAN-17-0357 PMC5679354 28904063

[241] Cao M Xu Y Youn JI Cabrera R Zhang X Gabrilovich D. Kinase inhibitor sorafenib modulates immunosuppressive cell populations in a murine liver cancer model. Lab Invest (2011) 91(4):598–608. doi: 10.1038/labinvest.2010.205 21321535 PMC3711234

[242] Husain Z Huang Y Seth P Sukhatme VP. Tumor-derived lactate modifies antitumor immune response: Effect on myeloid-derived suppressor cells and nk cells. J Immunol (2013) 191(3):1486–95. doi: 10.4049/jimmunol.1202702 23817426

[243] Tavazoie MF Pollack I Tanqueco R Ostendorf BN Reis BS Gonsalves FC. Lxr/Apoe activation restricts innate immune suppression in cancer. Cell (2018) 172(4):825–40 e18. doi: 10.1016/j.cell.2017.12.026 29336888 PMC5846344

[244] Liang H Shen X. Lxr activation radiosensitizes non-small cell lung cancer by restricting myeloid-derived suppressor cells. Biochem Biophys Res Commun (2020) 528(2):330–5. doi: 10.1016/j.bbrc.2020.04.137 32448508

[245] Allegrezza MJ Rutkowski MR Stephen TL Svoronos N Perales-Puchalt A Nguyen JM. Trametinib drives T-Cell-Dependent control of kras-mutated tumors by inhibiting pathological myelopoiesis. Cancer Res (2016) 76(21):6253–65. doi: 10.1158/0008-5472.CAN-16-1308 PMC5094194 27803104

[246] Baumann D Hagele T Mochayedi J Drebant J Vent C Blobner S. Proimmunogenic impact of mek inhibition synergizes with agonist anti-Cd40 immunostimulatory antibodies in tumor therapy. Nat Commun (2020) 11(1):2176. doi: 10.1038/s41467-020-15979-2 32358491 PMC7195409

[247] Schilling B Sucker A Griewank K Zhao F Weide B Gorgens A. Vemurafenib reverses immunosuppression by myeloid derived suppressor cells. Int J Cancer (2013) 133(7):1653–63. doi: 10.1002/ijc.28168 23526263

[248] Simpson KD Templeton DJ Cross JV. Macrophage migration inhibitory factor promotes tumor growth and metastasis by inducing myeloid-derived suppressor cells in the tumor microenvironment. J Immunol (2012) 189(12):5533–40. doi: 10.4049/jimmunol.1201161 PMC3518629 23125418

[249] Zhao T Du H Ding X Walls K Yan C. Activation of mtor pathway in myeloid-derived suppressor cells stimulates cancer cell proliferation and metastasis in lal(-/-) mice. Oncogene (2015) 34(15):1938–48. doi: 10.1038/onc.2014.143 PMC4254377 24882582

[250] Wu T Zhao Y Wang H Li Y Shao L Wang R. Mtor masters monocytic myeloid-derived suppressor cells in mice with allografts or tumors. Sci Rep (2016) 6:20250. doi: 10.1038/srep20250 26833095 PMC4735296

[251] Pi R Yang Y Hu X Li H Shi H Liu Y. Dual Mtorc1/2 inhibitor Azd2014 diminishes myeloid-derived suppressor cells accumulation in ovarian cancer and delays tumor growth. Cancer Lett (2021) 523:72–81. doi: 10.1016/j.canlet.2021.09.017 34560229

[252] Trikha P Plews RL Stiff A Gautam S Hsu V Abood D. Targeting myeloid-derived suppressor cells using a novel adenosine monophosphate-activated protein kinase (Ampk) activator. Oncoimmunology (2016) 5(9):e1214787. doi: 10.1080/2162402X.2016.1214787 27757311 PMC5048767

[253] Hong EH Chang SY Lee BR Kim YS Lee JM Kang CY. Blockade of Myd88 signaling induces antitumor effects by skewing the immunosuppressive function of myeloid-derived suppressor cells. Int J Cancer (2013) 132(12):2839–48. doi: 10.1002/ijc.27974 23184679

[254] Wang L Hu D Xie B Xie L. Blockade of Myd88 signaling by a novel Myd88 inhibitor prevents colitis-associated colorectal cancer development by impairing myeloid-derived suppressor cells. Invest New Drugs (2022) 40(3):506–18. doi: 10.1007/s10637-022-01218-6 PMC9098617 35089465

[255] Erin N Korcum AF Tanriover G Kale S Demir N Koksoy S. Activation of neuroimmune pathways increases therapeutic effects of radiotherapy on poorly differentiated breast carcinoma. Brain Behav Immun (2015) 48:174–85. doi: 10.1016/j.bbi.2015.02.024 25736062

[256] Kobayashi M Chung JS Beg M Arriaga Y Verma U Courtney K. Blocking monocytic myeloid-derived suppressor cell function *via* anti-Dc-Hil/Gpnmb antibody restores the in vitro integrity of T cells from cancer patients. Clin Cancer Res (2019) 25(2):828–38. doi: 10.1158/1078-0432.CCR-18-0330 PMC7315386 30049749

[257] Zonneville J Colligan S Grant S Miller A Wallace P Abrams SI. Blockade of P38 kinase impedes the mobilization of protumorigenic myeloid populations to impact breast cancer metastasis. Int J Cancer (2020) 147(8):2279–92. doi: 10.1002/ijc.33050 PMC7484223 32452014

[258] Alicea-Torres K Sanseviero E Gui J Chen J Veglia F Yu Q. Immune suppressive activity of myeloid-derived suppressor cells in cancer requires inactivation of the type I interferon pathway. Nat Commun (2021) 12(1):1717. doi: 10.1038/s41467-021-22033-2 33741967 PMC7979850

[259] Duraiswamy J Freeman GJ Coukos G. Therapeutic pd-1 pathway blockade augments with other modalities of immunotherapy T-cell function to prevent immune decline in ovarian cancer. Cancer Res (2013) 73(23):6900–12. doi: 10.1158/0008-5472.CAN-13-1550 PMC3851914 23975756

[260] John LB Devaud C Duong CP Yong CS Beavis PA Haynes NM. Anti-Pd-1 antibody therapy potently enhances the eradication of established tumors by gene-modified T cells. Clin Cancer Res (2013) 19(20):5636–46. doi: 10.1158/1078-0432.CCR-13-0458 23873688

[261] Noman MZ Desantis G Janji B Hasmim M Karray S Dessen P. Pd-L1 is a novel direct target of hif-1alpha, and its blockade under hypoxia enhanced mdsc-mediated T cell activation. J Exp Med (2014) 211(5):781–90. doi: 10.1084/jem.20131916 PMC4010891 24778419

[262] Yu GT Bu LL Huang CF Zhang WF Chen WJ Gutkind JS. Pd-1 blockade attenuates immunosuppressive myeloid cells due to inhibition of Cd47/Sirpalpha axis in hpv negative head and neck squamous cell carcinoma. Oncotarget (2015) 6(39):42067–80. doi: 10.18632/oncotarget.5955 PMC4747210 26573233

[263] Davis RJ Moore EC Clavijo PE Friedman J Cash H Chen Z. Anti-Pd-L1 efficacy can be enhanced by inhibition of myeloid-derived suppressor cells with a selective inhibitor of Pi3kdelta/Gamma. Cancer Res (2017) 77(10):2607–19. doi: 10.1158/0008-5472.CAN-16-2534 PMC5466078 28364000

[264] Teo ZL Versaci S Dushyanthen S Caramia F Savas P Mintoff CP. Combined Cdk4/6 and Pi3kalpha inhibition is synergistic and immunogenic in triple-negative breast cancer. Cancer Res (2017) 77(22):6340–52. doi: 10.1158/0008-5472.CAN-17-2210 28947417

[265] Lin H Wu Y Chen J Huang S Wang Y. (-)-4-O-(4-O-Beta-D-Glucopyranosylcaffeoyl) quinic acid inhibits the function of myeloid-derived suppressor cells to enhance the efficacy of anti-Pd1 against colon cancer. Pharm Res (2018) 35(9):183. doi: 10.1007/s11095-018-2459-5 30062658

[266] Zhang M Wang L Liu W Wang T De Sanctis F Zhu L. Targeting inhibition of accumulation and function of myeloid-derived suppressor cells by artemisinin *via* Pi3k/Akt, mtor, and mapk pathways enhances anti-Pd-L1 immunotherapy in melanoma and liver tumors. J Immunol Res (2022) 2022:2253436. doi: 10.1155/2022/2253436 35785030 PMC9247850

[267] Wu L Yan C Czader M Foreman O Blum JS Kapur R. Inhibition of ppargamma in myeloid-lineage cells induces systemic inflammation, immunosuppression, and tumorigenesis. Blood (2012) 119(1):115–26. doi: 10.1182/blood-2011-06-363093 PMC3251224 22053106

[268] Zhao T Du H Blum JS Yan C. Critical role of ppargamma in myeloid-derived suppressor cell-stimulated cancer cell proliferation and metastasis. Oncotarget (2016) 7(2):1529–43. doi: 10.18632/oncotarget.6414 PMC4811478 26625314

[269] Lu T Ramakrishnan R Altiok S Youn JI Cheng P Celis E. Tumor-infiltrating myeloid cells induce tumor cell resistance to cytotoxic T cells in mice. J Clin Invest (2011) 121(10):4015–29. doi: 10.1172/JCI45862 PMC3195459 21911941

[270] Yin Y Huang X Lynn KD Thorpe PE. Phosphatidylserine-targeting antibody induces M1 macrophage polarization and promotes myeloid-derived suppressor cell differentiation. Cancer Immunol Res (2013) 1(4):256–68. doi: 10.1158/2326-6066.CIR-13-0073 24777853

[271] Chalasani P Marron M Roe D Clarke K Iannone M Livingston RB. A phase I clinical trial of bavituximab and paclitaxel in patients with Her2 negative metastatic breast cancer. Cancer Med (2015) 4(7):1051–9. doi: 10.1002/cam4.447 PMC4529343 25826750

[272] Meyer C Sevko A Ramacher M Bazhin AV Falk CS Osen W. Chronic inflammation promotes myeloid-derived suppressor cell activation blocking antitumor immunity in transgenic mouse melanoma model. Proc Natl Acad Sci U.S.A. (2011) 108(41):17111–6. doi: 10.1073/pnas.1108121108 PMC3193202 21969559

[273] Umansky V Sevko A. Overcoming immunosuppression in the melanoma microenvironment induced by chronic inflammation. Cancer Immunol Immunother (2012) 61(2):275–82. doi: 10.1007/s00262-011-1164-6 PMC11028817 22120757

[274] Weed DT Vella JL Reis IM de la Fuente AC Gomez C Sargi Z. Tadalafil reduces myeloid-derived suppressor cells and regulatory T cells and promotes tumor immunity in patients with head and neck squamous cell carcinoma. Clin Cancer Res (2015) 21(1):39–48. doi: 10.1158/1078-0432.CCR-14-1711 25320361 PMC4322895

[275] Califano JA Khan Z Noonan KA Rudraraju L Zhang Z Wang H. Tadalafil augments tumor specific immunity in patients with head and neck squamous cell carcinoma. Clin Cancer Res (2015) 21(1):30–8. doi: 10.1158/1078-0432.CCR-14-1716 PMC4329916 25564570

[276] Lin S Wang J Wang L Wen J Guo Y Qiao W. Phosphodiesterase-5 inhibition suppresses colonic inflammation-induced tumorigenesis *Via* blocking the recruitment of mdsc. Am J Cancer Res (2017) 7(1):41–52. 28123846 PMC5250679

[277] Hassel JC Jiang H Bender C Winkler J Sevko A Shevchenko I. Tadalafil has biologic activity in human melanoma. results of a pilot trial with tadalafil in patients with metastatic melanoma (Tame). Oncoimmunology (2017) 6(9):e1326440. doi: 10.1080/2162402X.2017.1326440 28932631 PMC5599085

[278] Sinha P Clements VK Fulton AM Ostrand-Rosenberg S. Prostaglandin E2 promotes tumor progression by inducing myeloid-derived suppressor cells. Cancer Res (2007) 67(9):4507–13. doi: 10.1158/0008-5472.Can-06-4174 17483367

[279] Zhang Y Liu Q Zhang M Yu Y Liu X Cao X. Fas signal promotes lung cancer growth by recruiting myeloid-derived suppressor cells *Via* cancer cell-derived Pge2. J Immunol (2009) 182(6):3801–8. doi: 10.4049/jimmunol.0801548 19265159

[280] Obermajer N Muthuswamy R Odunsi K Edwards RP Kalinski P. Pge(2)-induced Cxcl12 production and Cxcr4 expression controls the accumulation of human mdscs in ovarian cancer environment. Cancer Res (2011) 71(24):7463–70. doi: 10.1158/0008-5472.Can-11-2449 PMC4993027 22025564

[281] Kidiyoor A Schettini J Besmer DM Rego SL Nath S Curry JM. Pancreatic cancer cells isolated from Muc1-null tumors favor the generation of a mature less suppressive mdsc population. Front Immunol (2014) 5:67. doi: 10.3389/fimmu.2014.00067 24605110 PMC3932420

[282] Mao Y Sarhan D Steven A Seliger B Kiessling R Lundqvist A. Inhibition of tumor-derived prostaglandin-E2 blocks the induction of myeloid-derived suppressor cells and recovers natural killer cell activity. Clin Cancer Res (2014) 20(15):4096–106. doi: 10.1158/1078-0432.Ccr-14-0635 24907113

[283] Blidner AG Salatino M Mascanfroni ID Diament MJ Bal de Kier Joffe E Jasnis MA. Differential response of myeloid-derived suppressor cells to the nonsteroidal anti-inflammatory agent indomethacin in tumor-associated and tumor-free microenvironments. J Immunol (2015) 194(7):3452–62. doi: 10.4049/jimmunol.1401144 25740944

[284] Guo Y Xiong J Wang J Wen J Zhi F. Inhibition of rac family protein impairs colitis and colitis-associated cancer in mice. Am J Cancer Res (2018) 8(1):70–80. 29416921 PMC5794722

[285] Strauss L Sangaletti S Consonni FM Szebeni G Morlacchi S Totaro MG. Rorc1 regulates tumor-promoting "Emergency" granulo-monocytopoiesis. Cancer Cell (2015) 28(2):253–69. doi: 10.1016/j.ccell.2015.07.006 26267538

[286] Nagaraj S Youn JI Weber H Iclozan C Lu L Cotter MJ. Anti-inflammatory triterpenoid blocks immune suppressive function of mdscs and improves immune response in cancer. Clin Cancer Res (2010) 16(6):1812–23. doi: 10.1158/1078-0432.CCR-09-3272 PMC2840181 20215551

[287] Alizadeh D Trad M Hanke NT Larmonier CB Janikashvili N Bonnotte B. Doxorubicin eliminates myeloid-derived suppressor cells and enhances the efficacy of adoptive T-cell transfer in breast cancer. Cancer Res (2014) 74(1):104–18. doi: 10.1158/0008-5472.CAN-13-1545 PMC3896092 24197130

[288] Ozao-Choy J Ma G Kao J Wang GX Meseck M Sung M. The novel role of tyrosine kinase inhibitor in the reversal of immune suppression and modulation of tumor microenvironment for immune-based cancer therapies. Cancer Res (2009) 69(6):2514–22. doi: 10.1158/0008-5472.CAN-08-4709 PMC4370269 19276342

[289] Holtzhausen A Harris W Ubil E Hunter DM Zhao J Zhang Y. Tam Family receptor kinase inhibition reverses mdsc-mediated suppression and augments anti-Pd-1 therapy in melanoma. Cancer Immunol Res (2019) 7(10):1672–86. doi: 10.1158/2326-6066.CIR-19-0008 PMC6943983 31451482

[290] Apolo AB Nadal R Tomita Y Davarpanah NN Cordes LM Steinberg SM. Cabozantinib in patients with platinum-refractory metastatic urothelial carcinoma: An open-label, single-centre, phase 2 trial. Lancet Oncol (2020) 21(8):1099–109. doi: 10.1016/S1470-2045(20)30202-3 PMC8236112 32645282

[291] Aggen DH Ager CR Obradovic AZ Chowdhury N Ghasemzadeh A Mao W. Blocking Il1 beta promotes tumor regression and remodeling of the myeloid compartment in a renal cell carcinoma model: Multidimensional analyses. Clin Cancer Res (2021) 27(2):608–21. doi: 10.1158/1078-0432.CCR-20-1610 PMC7980495 33148676

[292] Lu M Zhang X Gao X Sun S Wei X Hu X. Lenvatinib enhances T cell immunity and the efficacy of adoptive chimeric antigen receptor-modified T cells by decreasing myeloid-derived suppressor cells in cancer. Pharmacol Res (2021) 174:105829. doi: 10.1016/j.phrs.2021.105829 34411731

[293] Draghiciu O Nijman HW Hoogeboom BN Meijerhof T Daemen T. Sunitinib depletes myeloid-derived suppressor cells and synergizes with a cancer vaccine to enhance antigen-specific immune responses and tumor eradication. Oncoimmunology (2015) 4(3):e989764. doi: 10.4161/2162402X.2014.989764 25949902 PMC4404834

[294] Heine A Schilling J Grunwald B Kruger A Gevensleben H Held SA. The induction of human myeloid derived suppressor cells through hepatic stellate cells is dose-dependently inhibited by the tyrosine kinase inhibitors nilotinib, dasatinib and sorafenib, but not sunitinib. Cancer Immunol Immunother (2016) 65(3):273–82. doi: 10.1007/s00262-015-1790-5 PMC11029563 26786874

[295] Guislain A Gadiot J Kaiser A Jordanova ES Broeks A Sanders J. Sunitinib pretreatment improves tumor-infiltrating lymphocyte expansion by reduction in intratumoral content of myeloid-derived suppressor cells in human renal cell carcinoma. Cancer Immunol Immunother (2015) 64(10):1241–50. doi: 10.1007/s00262-015-1735-z PMC11028512 26105626

[296] Motoshima T Komohara Y Horlad H Takeuchi A Maeda Y Tanoue K. Sorafenib enhances the antitumor effects of anti-Ctla-4 antibody in a murine cancer model by inhibiting myeloid-derived suppressor cells. Oncol Rep (2015) 33(6):2947–53. doi: 10.3892/or.2015.3893 25845968

[297] Stiff A Trikha P Wesolowski R Kendra K Hsu V Uppati S. Myeloid-derived suppressor cells express bruton's tyrosine kinase and can be depleted in tumor-bearing hosts by ibrutinib treatment. Cancer Res (2016) 76(8):2125–36. doi: 10.1158/0008-5472.CAN-15-1490 PMC4873459 26880800

[298] Zhang T Harrison MR O'Donnell PH Alva AS Hahn NM Appleman LJ. A randomized phase 2 trial of pembrolizumab versus pembrolizumab and acalabrutinib in patients with platinum-resistant metastatic urothelial cancer. Cancer (2020) 126(20):4485–97. doi: 10.1002/cncr.33067 PMC7590121 32757302

[299] Overman M Javle M Davis RE Vats P Kumar-Sinha C Xiao L. Randomized phase ii study of the bruton tyrosine kinase inhibitor acalabrutinib, alone or with pembrolizumab in patients with advanced pancreatic cancer. J Immunother Cancer (2020) 8(1):e000587. doi: 10.1136/jitc-2020-000587 32114502 PMC7057435

[300] Varikuti S Singh B Volpedo G Ahirwar DK Jha BK Saljoughian N. Ibrutinib treatment inhibits breast cancer progression and metastasis by inducing conversion of myeloid-derived suppressor cells to dendritic cells. Br J Cancer (2020) 122(7):1005–13. doi: 10.1038/s41416-020-0743-8 PMC7109110 32025027

[301] Mao L Deng WW Yu GT Bu LL Liu JF Ma SR. Inhibition of src family kinases reduces myeloid-derived suppressor cells in head and neck cancer. Int J Cancer (2017) 140(5):1173–85. doi: 10.1002/ijc.30493 27798955

[302] Cheng P Corzo CA Luetteke N Yu B Nagaraj S Bui MM. Inhibition of dendritic cell differentiation and accumulation of myeloid-derived suppressor cells in cancer is regulated by S100a9 protein. J Exp Med (2008) 205(10):2235–49. doi: 10.1084/jem.20080132 PMC2556797 18809714

[303] Sinha P Okoro C Foell D Freeze HH Ostrand-Rosenberg S Srikrishna G. Proinflammatory S100 proteins regulate the accumulation of myeloid-derived suppressor cells. J Immunol (2008) 181(7):4666–75. doi: 10.4049/jimmunol.181.7.4666 PMC2810501 18802069

[304] Ichikawa M Williams R Wang L Vogl T Srikrishna G. S100a8/A9 activate key genes and pathways in colon tumor progression. Mol Cancer Res (2011) 9(2):133–48. doi: 10.1158/1541-7786.MCR-10-0394 PMC3078037 21228116

[305] Wang L Chang EW Wong SC Ong SM Chong DQ Ling KL. Increased myeloid-derived suppressor cells in gastric cancer correlate with cancer stage and plasma S100a8/A9 proinflammatory proteins. J Immunol (2013) 190(2):794–804. doi: 10.4049/jimmunol.1202088 23248262

[306] Ortiz ML Lu L Ramachandran I Gabrilovich DI. Myeloid-derived suppressor cells in the development of lung cancer. Cancer Immunol Res (2014) 2(1):50–8. doi: 10.1158/2326-6066.CIR-13-0129 PMC4007346 24778162

[307] Shen L Sundstedt A Ciesielski M Miles KM Celander M Adelaiye R. Tasquinimod modulates suppressive myeloid cells and enhances cancer immunotherapies in murine models. Cancer Immunol Res (2015) 3(2):136–48. doi: 10.1158/2326-6066.CIR-14-0036 PMC4323929 25370534

[308] Deguchi A Tomita T Ohto U Takemura K Kitao A Akashi-Takamura S. Eritoran inhibits S100a8-mediated Tlr4/Md-2 activation and tumor growth by changing the immune microenvironment. Oncogene (2016) 35(11):1445–56. doi: 10.1038/onc.2015.211 26165843

[309] Li H Han Y Guo Q Zhang M Cao X. Cancer-expanded myeloid-derived suppressor cells induce anergy of nk cells through membrane-bound tgf-beta 1. J Immunol (2009) 182(1):240–9. doi: 10.4049/jimmunol.182.1.240 19109155

[310] Korbelik M Banath J Zhang W Saw KM Szulc ZM Bielawska A. Interaction of acid ceramidase inhibitor Lcl521 with tumor response to photodynamic therapy and photodynamic therapy-generated vaccine. Int J Cancer (2016) 139(6):1372–8. doi: 10.1002/ijc.30171 27136745

[311] Liu F Li X Lu C Bai A Bielawski J Bielawska A. Ceramide activates lysosomal cathepsin b and cathepsin d to attenuate autophagy and induces er stress to suppress myeloid-derived suppressor cells. Oncotarget (2016) 7(51):83907–25. doi: 10.18632/oncotarget.13438 PMC5356634 27880732

[312] Plebanek MP Bhaumik D Bryce PJ Thaxton CS. Scavenger receptor type B1 and lipoprotein nanoparticle inhibit myeloid-derived suppressor cells. Mol Cancer Ther (2018) 17(3):686–97. doi: 10.1158/1535-7163.MCT-17-0981 PMC5935575 29282300

[313] Younis RH Han KL Webb TJ. Human head and neck squamous cell carcinoma-associated semaphorin 4d induces expansion of myeloid-derived suppressor cells. J Immunol (2016) 196(3):1419–29. doi: 10.4049/jimmunol.1501293 PMC4722498 26740106

[314] Clavijo PE Friedman J Robbins Y Moore EC Smith E Zauderer M. Semaphorin4d inhibition improves response to immune-checkpoint blockade *Via* attenuation of mdsc recruitment and function. Cancer Immunol Res (2019) 7(2):282–91. doi: 10.1158/2326-6066.CIR-18-0156 PMC8326929 30514791

[315] Liu G Bi Y Shen B Yang H Zhang Y Wang X. Sirt1 limits the function and fate of myeloid-derived suppressor cells in tumors by orchestrating hif-1alpha-Dependent glycolysis. Cancer Res (2014) 74(3):727–37. doi: 10.1158/0008-5472.CAN-13-2584 24351289

[316] Xin H Zhang C Herrmann A Du Y Figlin R Yu H. Sunitinib inhibition of Stat3 induces renal cell carcinoma tumor cell apoptosis and reduces immunosuppressive cells. Cancer Res (2009) 69(6):2506–13. doi: 10.1158/0008-5472.CAN-08-4323 PMC2664264 19244102

[317] Poschke I Mougiakakos D Hansson J Masucci GV Kiessling R. Immature immunosuppressive Cd14+Hla-Dr-/Low cells in melanoma patients are Stat3hi and overexpress Cd80, Cd83, and dc-sign. Cancer Res (2010) 70(11):4335–45. doi: 10.1158/0008-5472.CAN-09-3767 20484028

[318] Tu SP Jin H Shi JD Zhu LM Suo Y Lu G. Curcumin induces the differentiation of myeloid-derived suppressor cells and inhibits their interaction with cancer cells and related tumor growth. Cancer Prev Res (Phila) (2012) 5(2):205–15. doi: 10.1158/1940-6207.CAPR-11-0247 PMC3273601 22030090

[319] Vasquez-Dunddel D Pan F Zeng Q Gorbounov M Albesiano E Fu J. Stat3 regulates arginase-I in myeloid-derived suppressor cells from cancer patients. J Clin Invest (2013) 123(4):1580–9. doi: 10.1172/JCI60083 PMC3613901 23454751

[320] Panni RZ Sanford DE Belt BA Mitchem JB Worley LA Goetz BD. Tumor-induced Stat3 activation in monocytic myeloid-derived suppressor cells enhances stemness and mesenchymal properties in human pancreatic cancer. Cancer Immunol Immunother (2014) 63(5):513–28. doi: 10.1007/s00262-014-1527-x PMC3994288 24652403

[321] Yu J Wang Y Yan F Zhang P Li H Zhao H. Noncanonical nf-kappab activation mediates Stat3-stimulated ido upregulation in myeloid-derived suppressor cells in breast cancer. J Immunol (2014) 193(5):2574–86. doi: 10.4049/jimmunol.1400833 PMC4719564 25063873

[322] Yang F Hu M Lei Q Xia Y Zhu Y Song X. Nifuroxazide induces apoptosis and impairs pulmonary metastasis in breast cancer model. Cell Death Dis (2015) 6:e1701. doi: 10.1038/cddis.2015.63 25811798 PMC4385941

[323] Zhu Y Ye T Yu X Lei Q Yang F Xia Y. Nifuroxazide exerts potent anti-tumor and anti-metastasis activity in melanoma. Sci Rep (2016) 6:20253. doi: 10.1038/srep20253 26830149 PMC4735744

[324] Ye TH Yang FF Zhu YX Li YL Lei Q Song XJ. Inhibition of Stat3 signaling pathway by nifuroxazide improves antitumor immunity and impairs colorectal carcinoma metastasis. Cell Death Dis (2017) 8(1):e2534. doi: 10.1038/cddis.2016.452 28055016 PMC5386364

[325] Al-Khami AA Zheng L Del Valle L Hossain F Wyczechowska D Zabaleta J. Exogenous lipid uptake induces metabolic and functional reprogramming of tumor-associated myeloid-derived suppressor cells. Oncoimmunology (2017) 6(10):e1344804. doi: 10.1080/2162402X.2017.1344804 29123954 PMC5665069

[326] Bitsch R Kurzay A Ozbay Kurt F de la Torre C Lasser S Lepper A. Stat3 inhibitor napabucasin abrogates mdsc immunosuppressive capacity and prolongs survival of melanoma-bearing mice. J Immunother Cancer (2022) 10(3):e004384. doi: 10.1136/jitc-2021-004384 35301236 PMC8932276

[327] Liu D You M Xu Y Li F Zhang D Li X. Inhibition of curcumin on myeloid-derived suppressor cells is requisite for controlling lung cancer. Int Immunopharmacol (2016) 39:265–72. doi: 10.1016/j.intimp.2016.07.035 27497194

[328] Tian S Liao L Zhou Q Huang X Zheng P Guo Y. Curcumin inhibits the growth of liver cancer by impairing myeloid-derived suppressor cells in murine tumor tissues. Oncol Lett (2021) 21(4):286. doi: 10.3892/ol.2021.12547 33732362 PMC7905673

[329] Wang SH Lu QY Guo YH Song YY Liu PJ Wang YC. The blockage of notch signalling promoted the generation of polymorphonuclear myeloid-derived suppressor cells with lower immunosuppression. Eur J Cancer (2016) 68:90–105. doi: 10.1016/j.ejca.2016.08.019 27728841

[330] Mao L Zhao ZL Yu GT Wu L Deng WW Li YC. Gamma-secretase inhibitor reduces immunosuppressive cells and enhances tumour immunity in head and neck squamous cell carcinoma. Int J Cancer (2018) 142(5):999–1009. doi: 10.1002/ijc.31115 29047105

[331] Qiu H Zmina PM Huang AY Askew D Bedogni B. Inhibiting Notch1 enhances immunotherapy efficacy in melanoma by preventing Notch1 dependent immune suppressive properties. Cancer Lett (2018) 434:144–51. doi: 10.1016/j.canlet.2018.07.024 PMC7185871 30036609

[332] Zhang CX Ye SB Ni JJ Cai TT Liu YN Huang DJ. Sting signaling remodels the tumor microenvironment by antagonizing myeloid-derived suppressor cell expansion. Cell Death Differ (2019) 26(11):2314–28. doi: 10.1038/s41418-019-0302-0 PMC6889506 30816302

[333] Wang G Zhou X Guo Z Huang N Li J Lv Y. The anti-fibrosis drug pirfenidone modifies the immunosuppressive tumor microenvironment and prevents the progression of renal cell carcinoma by inhibiting tumor autocrine tgf-beta. Cancer Biol Ther (2022) 23(1):150–62. doi: 10.1080/15384047.2022.2035629 PMC8824226 35130111

[334] Gobbo J Marcion G Cordonnier M Dias AMM Pernet N Hammann A. Restoring anticancer immune response by targeting tumor-derived exosomes with a Hsp70 peptide aptamer. J Natl Cancer Inst (2016) 108(3):djv330. doi: 10.1093/jnci/djv330 26598503

[335] Deng Y Yang J Qian J Liu R Huang E Wang Y. Tlr1/Tlr2 signaling blocks the suppression of monocytic myeloid-derived suppressor cell by promoting its differentiation into M1-type macrophage. Mol Immunol (2019) 112:266–73. doi: 10.1016/j.molimm.2019.06.006 31212097

[336] Zhang W He W Shi X Li X Wang Y Hu M. An asparagus polysaccharide fraction inhibits mdscs by inducing apoptosis through toll-like receptor 4. Phytother Res (2018) 32(7):1297–303. doi: 10.1002/ptr.6058 29532545

[337] He W Zhang W Zheng Q Wei Z Wang Y Hu M. Cinnamaldehyde causes apoptosis of myeloid-derived suppressor cells through the activation of Tlr4. Oncol Lett (2019) 18(3):2420–6. doi: 10.3892/ol.2019.10544 PMC6676727 31402944

[338] Shayan G Kansy BA Gibson SP Srivastava RM Bryan JK Bauman JE. Phase ib study of immune biomarker modulation with neoadjuvant cetuximab and Tlr8 stimulation in head and neck cancer to overcome suppressive myeloid signals. Clin Cancer Res (2018) 24(1):62–72. doi: 10.1158/1078-0432.CCR-17-0357 29061643 PMC5754237

[339] Dang Y Rutnam ZJ Dietsch G Lu H Yang Y Hershberg R. Tlr8 ligation induces apoptosis of monocytic myeloid-derived suppressor cells. J Leukoc Biol (2018) 103(1):157–64. doi: 10.1002/JLB.5AB0217-070R PMC6721845 29345064

[340] Cho JH Lee HJ Ko HJ Yoon BI Choe J Kim KC. The Tlr7 agonist imiquimod induces anti-cancer effects *via* autophagic cell death and enhances anti-tumoral and systemic immunity during radiotherapy for melanoma. Oncotarget (2017) 8(15):24932–48. doi: 10.18632/oncotarget.15326 PMC5421900 28212561

[341] Spinetti T Spagnuolo L Mottas I Secondini C Treinies M Ruegg C. Tlr7-based cancer immunotherapy decreases intratumoral myeloid-derived suppressor cells and blocks their immunosuppressive function. Oncoimmunology (2016) 5(11):e1230578. doi: 10.1080/2162402X.2016.1230578 27999739 PMC5139641

[342] Liu Z Xie Y Xiong Y Liu S Qiu C Zhu Z. Tlr 7/8 agonist reverses oxaliplatin resistance in colorectal cancer *Via* directing the myeloid-derived suppressor cells to tumoricidal M1-macrophages. Cancer Lett (2020) 469:173–85. doi: 10.1016/j.canlet.2019.10.020 31629935

[343] Zoglmeier C Bauer H Noerenberg D Wedekind G Bittner P Sandholzer N. Cpg blocks immunosuppression by myeloid-derived suppressor cells in tumor-bearing mice. Clin Cancer Res (2011) 17(7):1765–75. doi: 10.1158/1078-0432.CCR-10-2672 21233400

[344] Hossain DM Pal SK Moreira D Duttagupta P Zhang Q Won H. Tlr9-targeted Stat3 silencing abrogates immunosuppressive activity of myeloid-derived suppressor cells from prostate cancer patients. Clin Cancer Res (2015) 21(16):3771–82. doi: 10.1158/1078-0432.CCR-14-3145 PMC4537814 25967142

[345] Sobo-Vujanovic A Vujanovic L DeLeo AB Concha-Benavente F Ferris RL Lin Y. Inhibition of soluble tumor necrosis factor prevents chemically induced carcinogenesis in mice. Cancer Immunol Res (2016) 4(5):441–51. doi: 10.1158/2326-6066.CIR-15-0104 PMC4873325 26896171

[346] Atretkhany KS Nosenko MA Gogoleva VS Zvartsev RV Qin Z Nedospasov SA. Tnf neutralization results in the delay of transplantable tumor growth and reduced mdsc accumulation. Front Immunol (2016) 7:147. doi: 10.3389/fimmu.2016.00147 27148266 PMC4835443

[347] Dominguez GA Condamine T Mony S Hashimoto A Wang F Liu Q. Selective targeting of myeloid-derived suppressor cells in cancer patients using ds-8273a, an agonistic trail-R2 antibody. Clin Cancer Res (2017) 23(12):2942–50. doi: 10.1158/1078-0432.CCR-16-1784 PMC5468499 27965309

[348] Hartwig T Montinaro A von Karstedt S Sevko A Surinova S Chakravarthy A. The trail-induced cancer secretome promotes a tumor-supportive immune microenvironment *Via* Ccr2. Mol Cell (2017) 65(4):730–42 e5. doi: 10.1016/j.molcel.2017.01.021 28212753 PMC5316415

[349] Hiramoto K Satoh H Suzuki T Moriguchi T Pi J Shimosegawa T. Myeloid lineage-specific deletion of antioxidant system enhances tumor metastasis. Cancer Prev Res (Phila) (2014) 7(8):835–44. doi: 10.1158/1940-6207.CAPR-14-0094 24866179

[350] Espagnolle N Barron P Mandron M Blanc I Bonnin J Agnel M. Specific inhibition of the vegfr-3 tyrosine kinase by Sar131675 reduces peripheral and tumor associated immunosuppressive myeloid cells. Cancers (Basel) (2014) 6(1):472–90. doi: 10.3390/cancers6010472 PMC3980599 24589997

[351] Feng PH Chen KY Huang YC Luo CS Wu SM Chen TT. Bevacizumab reduces S100a9-positive mdscs linked to intracranial control in patients with egfr-mutant lung adenocarcinoma. J Thorac Oncol (2018) 13(7):958–67. doi: 10.1016/j.jtho.2018.03.032 29684573

[352] Maughan BL Pal SK Gill D Boucher K Martin C Salgia M. Modulation of premetastatic niche by the vascular endothelial growth factor receptor tyrosine kinase inhibitor pazopanib in localized high-risk prostate cancer followed by radical prostatectomy: A phase ii randomized trial. Oncologist (2018) 23(12):1413–e151. doi: 10.1634/theoncologist.2018-0652 30575560 PMC6292560

[353] Horikawa N Abiko K Matsumura N Baba T Hamanishi J Yamaguchi K. Anti-vegf therapy resistance in ovarian cancer is caused by gm-Csf-Induced myeloid-derived suppressor cell recruitment. Br J Cancer (2020) 122(6):778–88. doi: 10.1038/s41416-019-0725-x PMC7078258 31932754

[354] Lacal PM Atzori MG Ruffini F Scimeca M Bonanno E Cicconi R. Targeting the vascular endothelial growth factor receptor-1 by the monoclonal antibody D16f7 to increase the activity of immune checkpoint inhibitors against cutaneous melanoma. Pharmacol Res (2020) 159:104957. doi: 10.1016/j.phrs.2020.104957 32485280

[355] Grover A Sanseviero E Timosenko E Gabrilovich DI. Myeloid-derived suppressor cells: A propitious road to clinic. Cancer Discov (2021) 11(11):2693–706. doi: 10.1158/2159-8290.CD-21-0764 34635571

[356] Veglia F Sanseviero E Gabrilovich DI. Myeloid-derived suppressor cells in the era of increasing myeloid cell diversity. Nat Rev Immunol (2021) 21(8):485–98. doi: 10.1038/s41577-020-00490-y PMC7849958 33526920

[357] Jensen KP Hongo DA Ji X Zheng P Pawar R Wu TH. Development of immunosuppressive myeloid cells to induce tolerance in solid organ and hematopoietic cell transplant recipients. Blood Adv (2021) 5(17):3290–302. doi: 10.1182/bloodadvances.2020003669 PMC8525233 34432869

